# Findings Favor Haptics Feedback in Virtual Simulation Surgical Education: An Updated Systematic and Scoping Review

**DOI:** 10.1177/15533506241238263

**Published:** 2024-03-14

**Authors:** Sayed Azher, Aralia Mills, Jinzhi He, Taliah Hyjazie, Junko Tokuno, Andrea Quaiattini, Jason M. Harley

**Affiliations:** 1 Department of Surgery, 12367 McGill University, Montreal, QC, Canada; 2 Simulation, Affect, Innovation, Learning, and Surgery (SAILS) Lab, Faculty of Medicine and Health Sciences, 12367 McGill University, Montreal, QC, Canada; 3 Steinberg Centre for Simulation and Interactive Learning, Faculty of Medicine and Health Sciences, 12367 McGill University, Montreal, QC, Canada; 4 Institute of Health Sciences Education, Faculty of Medicine and Health Sciences, 12367 McGill University, Montreal, QC, Canada; 5 Schulich Library of Physical Sciences, Life Sciences, and Engineering, 12367 McGill University, Montreal, QC, Canada; 6 Research Institute of the McGill University Health Centre, Montreal, QC, Canada

**Keywords:** virtual simulation, haptic, force feedback, simulation, surgery, training

## Abstract

**Background:**

Virtual simulations (VSs) enhance clinical competencies and skills. However, a previous systematic review of 9 RCT studies highlighted a paucity of literature on the effects of haptic feedback in surgical VSs. An updated systematic and scoping review was conducted to encompass more studies and a broader range of study methodologies.

**Methods:**

A systematic literature search was conducted on July 31, 2023, in MEDLINE, Embase, and Cochrane. English language studies comparing haptic vs non-haptic conditions and using VSs were included. Studies were evaluated and reported using PRISMA-ScR guidelines.

**Results:**

Out of 2782 initial studies, 51 were included in the review. Most studies used RCT (21) or crossover (23) methodologies with medical residents, students, and attending physicians. Most used post-intervention metrics, while some used pre- and post-intervention metrics. Overall, 34 performance results from studies favored haptics, 3 favored non-haptics, and the rest showed mixed or equal results.

**Conclusion:**

This updated review highlights the diverse application of haptic technology in surgical VSs. Haptics generally enhances performance, complements traditional teaching methods, and offers personalized learning with adequate simulator validation. However, a sparsity of orienting to the simulator, pre-/post-study designs, and small sample sizes poses concerns with the validity of the results. We underscore the urgent need for standardized protocols, large-scale studies, and nuanced understanding of haptic feedback integration. We also accentuate the significance of simulator validation, personalized learning potential, and the need for researcher, educator, and manufacturer collaboration. This review is a guidepost for navigating the complexities and advancements in haptic-enhanced surgical VSs.

## Introduction

Healthcare education has transitioned from conventional apprenticeship-based to a more simulation-focused model of teaching.^1–7^ This more recent model allows healthcare learners to freely learn from mistakes and apply theoretical knowledge before treating patients in real-life.^1–7^ Simulations can occur either in-person or virtually.^1,8^ In-person simulations involve role-playing individuals (eg, standardized patient actors) and manikins.^1,8^ In-person simulations thus require human resources and equipment which can be very expensive.^1,8^ An alternative to in-person simulations are virtual simulations (VSs), which use computer hardware and software to achieve the same goal.^8,9^ VSs provide a game-like environment where students can complete educational scenarios involving virtual patients or organs, similar in content to in-person simulations.^10^

Virtual simulations are effective in teaching students and can improve clinical competencies,^11^ declarative knowledge,^12^ procedural skills,^13,14^ and clinical decision-making.^15^ However, VS technology is still emerging with new features being added to replicate realism.^9^ A large limitation in many VSs is the lack of force feedback (eg, haptics).^1,9^ To guide our research, we define haptics as force feedback or any feedback that is delivered in vibrational form.^16^ The perception of haptics includes both tactile and somatosensory perception via the skin as well as kinesthetic proprioceptive feedback in other body parts (eg, muscles and ligaments).^17^ Haptics are thought of as being crucial in VSs, especially those relating to surgery as safe tissue handling and motor skill acquisition rely on haptic cues.^16,18–21^

With the safer preference of laparoscopic, robotics, and minimally invasive surgical procedures over traditional surgical intervention,^22^ there is a clear need for surgical training that replicates realism during learning to ensure transferability of ex vivo skills to real-life situations.^23^ As such, research on haptics is seen as essential, yet rarely assessed/incorporated in VSs.^23^ This is evident as a prominent systematic review of haptics in VSs found only nine total studies to include in their review.^23^ To the author’s knowledge, this review represents the sole review-based investigation comparing haptics vs non-haptic conditions in VSs. While the review by Rangarajan and colleagues^23^ was a crucial and informative review, its search is limited up to 2019, which at the time was recent; however, many advances have been made regarding haptic integrations within VSs since. The rapid evolution of haptic technology necessitates the need to conduct an updated review.^24^ Furthermore, the previous review focused primarily on randomized controlled trials (RCTs), and thus not capturing a wide array of studies within surgical education with other methodologies.^23^ Small sample sizes due to specialized populations (eg, medical residents and physicians) are common in this field, and accordingly, many studies are unable to pursue an RCT methodology. As such, we argue that focusing on RCTs excludes many relevant studies and does not give a comprehensive picture of the research landscape. Consequently, an updated review is needed to include findings from all study types and include the plethora of studies that have proliferated since, including but not limited to RCTs.

Therefore, we conducted an updated scoping review using a systematic methodology to assess the evidence in the literature regarding the use of haptics within educational VSs. Towards this end, we were interested in the methodologies employed by studies, their use of simulators, and evidence for the use of haptics in VSs.

## Material and Methods

### Search Strategy

A scoping review was conducted in accordance with Arksey and O’Malley’s framework^25^ and is reported using the Preferred Reporting Items for Systematic Reviews and Meta-Analyses Extension for Scoping Reviews (PRISMA-ScR) guidelines.^26^ The literature search was conducted by a health sciences librarian (AQ) to identify all relevant literature examining the use of haptics in virtual surgical simulations. The search strategy for the review was informed by the previous review as the previous review’s search was not reproducible.^23^ The databases searched include Ovid MEDLINE, Ovid Embase, and Cochrane Central Register of Controlled Trials. The search period covered the databases’ inception to 31 July 2023,^23^ (see supplemental material for complete search strategies).

### Data Extraction and Selection Criteria

The results were compiled from the databases and duplicates were removed in EndNote 20 (Build 16860)^27^ and Microsoft Excel.^28^

After obtaining a list of studies from the search strategy, six reviewers were involved in blinded title/abstract and full-text screening processes. In both stages, pilot screenings were performed to mitigate any conflicts. Final screenings at each stage began once the pilot stage had achieved 80% agreement. Screening was conducted using Microsoft Excel.^28^ After full-text screening, a data extraction chart was developed in Microsoft Excel and extraction included five reviewers (one senior and four junior reviewers). Junior reviewers formed 2 pairs. Independent calibration was also conducted prior to data extraction, and discrepancies were resolved for all reviewers by the senior reviewer. Once reaching sufficient agreement (at least 80%),^29^ an additional pilot extraction was performed between the senior reviewer and both pairs of junior reviewers. The agreement was once again sufficient (above 80%),^29^ and remaining data extraction was conducted by the two pairs of junior reviewers. Similar to the previous review,^23^ a meta-analysis methodology was not undertaken due to the extensive heterogeneity of the data and methodologies. While studies could be grouped by experimental design, there existed considerable variability in terms of haptic devices, software choice, and metrics.

We included studies that: (1) were in English, (2) involved a VS, and (3) compared a haptic condition to a non-haptic condition. We excluded studies that (1) were in another language than English, (2) did not involve a VS, or (3) did not have a haptic-enabled and haptic-disabled condition/comparison. Gray literature (eg, conference papers) were also included if that had sufficient data and elaboration of results.

### Data Synthesis and Study Assessment

Data were synthesized in a 3-step process: qualitative analysis, reporting of findings, and discussion of relevant results/conclusions.^30,31^ The findings from each study were evaluated by a senior reviewer with research experience and a junior reviewer with medical expertise (undergraduate medical student). The purpose of this evaluation is to provide a broad summative overview for the readers. Study quality was also assessed systematically for all included studies. The medical education research study quality instrument (MERSQI) was utilized to assess all included studies due to its broad applicability to various study designs.^32^ The Critical Appraisal Skills Programme (CASP) RCT Standard Checklist^33^ and The Joanna Briggs Institute (JBI) Critical Appraisal Checklist for Quasi-Experimental Studies^34^ were also utilized to assess RCTs and experimental study designs, respectively.

## Results

A total of 2782 potentially relevant studies were yielded in the initial search. After deduplication, 1707 studies remained. Following abstract and full-text screening, 51 were included for data extraction and synthesis (Figure 1).^20,35–84^

### Search Results and Study Characteristics

Of the 51 studies, our search captured all 9 studies in the previous review.^20,35,39,43–46,48,64^ Of the remaining 42 studies, 30 were from 2019 or earlier and 12 were new and relevant studies (Table 1). Crossover methodologies were employed most frequently (23 studies, 45.10%) followed by RCTs (21 studies, 41.18%). The remaining studies utilized quasi-experimental methodologies (7 studies, 13.73%). Notably, our review found 17 RCTs^38,41,49,50,54,55,58,65,67–69,77,78,81–84^ that were not included in the previous review, 11 of which were published before 2019^38,41,49,50,54,55,58,65,67–69^ and the rest being novel studies.^77,78,81–84^

The most common population sampled were medical residents (14 studies), followed by novices (9 studies), physicians (8 studies), and medical students (7 studies). A minority of studies collected data from dentistry students (3 studies), and patients (2 studies). The remaining 8 studies sampled across a mosaic of populations (eg, medical students + physicians + biomedical scientists). Notably, studies describing their sample as “novices” did not provide any further information regarding participant classification (eg, medical resident, fellow or medical students). The mean sample size across studies was 24 (1 gray literature study did not explicitly report its sample size),^67^ though sample sizes ranged from 1 to 79.

Most studies drew comparisons between haptics and non-haptic conditions by utilizing only post-intervention (29 studies, 56.86%) metrics, while 18 studies (35.29%) used both pre- and post-intervention metrics. A minority of studies (4 studies, 7.84%) conducted assessment of metrics over multiple days. Relatedly, prior to beginning data collection, 22 studies (43.14%) studies gave students oral instructions and allocated time for practice on the respective simulation device (eg, technological orientation). 10 studies (19.61%) gave only oral instructions while 7 studies (13.73%) only provided hands-on practice. 12 studies (23.53%) did not provide any technological orientation to participants.

### Relevant Results from Included Studies

A variety of simulators were used across studies with the most used being the LapSim platform (6 studies). Other studies either built their own simulators with haptic feedback using Phantom devices (eg, a device with a handle attached to a mobile arm that participants can manipulate and feel haptic feedback)^52^ and/or utilized other VS platforms such as Lap Mentor and ProMIS. The assessed haptic and non-haptic tasks varied by studies but broadly fell into the following categories: surgical tasks (n = 101), dentistry-specific tasks (n = 31), and non-surgical tasks (n = 12). Surgical tasks included basics tasks such as tool insertion attempts, movement distance, camera angle manipulation, as well as complex tasks including economy of hand movement, and suturing. Dentistry-specific tasks also had basic and complex tasks such as mean drilling depth and mean buccolingual angle deviation, respectively. Lastly, non-surgical tasks consisted predominantly of survey-based metrics regarding the perceptions of the haptic-compatible simulator (eg, preference for haptic vs non-haptic environment). An in-depth presentation of all assessed tasks is presented in the supplemental material while a general summary is provided in Table 2.

The characteristics of haptics (eg, the sense that devices or simulators provided) were not elaborated on in twenty-four studies (n = 24). Among the studies that described these details, sense of collision or touch (n = 12),^39,40,51,52,60–62,70,71,75,77,85^ drilling (n = 7),^49,58,59,64,78,82,83^ and force changes due to interactions with different tissue types (eg, skin, adipose tissue, vessels, n = 4)^50,68,69,74^ were the replicated haptic interactions. In two studies (n = 2),^47,65^ haptics were used to provide guidance to participants to operate the haptic robot within a predefined path. Among studies that specified mechanical aspects of the device and delivery of haptics, the majority utilized haptic arms (n = 23) or laparoscopic simulators (eg, LapSim, Lap Mentor II; n = 10). In studies utilizing haptic arms, the most common device was the Phantom Omni arm which consisted of a mobile arm with six degrees of freedom that the participants could manipulate and feel haptic feedback generated via a control box.^52^ In laparoscopic simulator, instrument handles^41^ or trocars^86^ were utilized to exert the haptic force. To replicate the multisensory nature of surgical practice,^61^ in addition to haptic, reinforcement of visual information (eg, shadows of instruments, tissue deformation) was added to enhance realism (n = 19).^35,36,40,41,47,51,56,59–61,64,65,67,70,71,73,77,78,86^ Sound effects during collisions and drilling were also reported to enhance realism (n = 8).^49,60,71,75–77,82,83^

### Studies Favoring Haptics (n = 34)

34 studies had results that, overall, favored the haptic condition over non-haptic while the summative results of only 3 studies^59,66,86^ favored the non-haptic condition. The remaining studies either showed the two conditions to be equal/similar (n = 11)^39,42,44,45,50,54,67,74,77,80,83^ or had mixed results (n = 3)^52,68,81^ such that some assessed variables favored the haptic condition while others favored non-haptics. The important results from included studies are summarized in Table 1 while a more nuanced picture of each task and the condition it favored is listed in Table 3.

Studies showcasing favorable outcomes for the haptic condition tended to display a variety of benefits in different aspects. For one, time to complete tasks and basic instrument handling tasks were often seen as being better in the haptic condition. Furthermore, the general trend across studies was that haptics helped students reach proficiency faster relative to non-haptics. Other general trends often yielded positive subjective ratings of haptic feedback and optimistic outlook regarding its integration into training, especially for fundamental tasks. These results are not unexpected as the previous review highlights many of the same, albeit with a much smaller number of studies (9 vs 51).

### Studies With Mixed Results (n = 3)

Three studies demonstrated mixed results which favored the haptic condition only under certain aspects. Vamadevan and colleagues tasked residents to reach proficiency in either a haptic or non-haptic simulator (intervention) and then reach proficiency in the non-haptic simulator (3-6 week follow-up).^81^ The haptic group required less instructor assistance and reached proficiency faster during the intervention, while the non-haptic group reached proficiency faster during the follow-up test.^81^ Across both phases, the haptic group required significantly more instructor feedback during the follow-up than intervention while the non-haptic group required significantly less.^81^ The authors concluded that acquired skills were not transferable to a conventional non-haptic setting.^81^ Mixed results in terms of errors were demonstrated by Yovanoff and colleagues who assigned medical residents to either use a manikin (non-haptic) or a haptic robotic simulator to learn internal jugular central venous catheterization training followed by feedback from either an upper-level resident (manikin) or the simulator.^68^ Results from pre- and post-tests showed that both groups improved their self-efficacy scores and the non-haptic group consistently had higher self-rating of performance.^68^ However, the simulator group had significantly more feedback instances and their self-ratings of performance significantly predicted the simulator’s given score.^68^ Interestingly, the haptic group had a significantly greater number of errors in general procedural steps while the non-haptic group had significantly more errors relating to pressure or torque applied.^68^ Lastly, mixed results between objective and subjective outcomes were shown by LeBlanc and colleagues who tasked orthopedic residents with performing a surgical fixation of the ulna on either a virtual haptic simulator or sawbones (non-haptic) followed by the same procedure on the other simulator.^52^ Their performance was evaluated by examiners via a task-specific checklist, global rating scale, and time-to-completion.^52^ Residents in the haptic group performed significantly better in both the checklist score and global rating scale score.^52^ However, the haptic group took significantly longer (by approximately a minute) to finish the procedure.^52^ Residents found that the virtual simulator needed further improvement and that they would prefer the sawbones (non-haptic) simulator if given the choice.^52^

### Studies Favoring Non-haptics (n = 3)

Three studies identified negative results of haptic feedback in VSs. Hochman and colleagues compared printed temporal bone model (non-haptic) to a virtual haptic graphic surgical simulation of a similar model (haptic condition).^59^ Participants found several mechanical properties of the physical bone model to be more comparable to a cadaveric bone than the virtual model.^59^ There were no differences between models in anatomic features and both models were found to be productive resources for acquiring surgical skills.^59^ While both conditions were rated similarly in terms of ease of use and a visual learning tool, the physical non-haptic model was ranked more effective for learning and considered the superior tool in other domains (eg, visual realism, bonelike properties, etc.).^59^ Furthermore, a survey study conducted by Vapenstad and colleagues (2013) compared the opinions of surgeons with different levels of laparoscopic experience regarding two VS instrument ports: one with haptics, and another without.^86^ While most surgeons said that haptic feedback was important, most thought that the non-haptic instrument port felt more realistic.^86^ The haptic port was seen as having too high friction and most surgeons performed best without it.^86^ Notably, the simulator was deemed to be the issue in the study.^86^ A subsequent study by Vapenstad and colleagues (2017) trained participants either in a simulator group (with haptic feedback) or control (no training, non-haptics) prior to performing a cholecystectomy in a box trainer.^66^ Video assessment of participants’ performance showed better performance of the non-haptic group in perception, bimanual dexterity, and efficiency.^66^ Tissue handling was not different between conditions.^66^ However, the authors noted, once again, that this negative training effect may be attributed to the poor mechanical performance of the simulator utilized.^66^

### Assessment of Methodological Quality

The mean MERSQI score across all papers was 12.82 (1.59). This score represents a moderate rating across studies.^32^ The lowest scored categories were related to sample size and validity of the evaluation instruments utilized. Scores for all categories are shown in Table 4. Results from the CASP RCT standard checklist and JBI critical appraisal checklist for quasi-experimental studies are shown in Tables 5 and 6, respectively.

## Discussion

This new and more comprehensive review was an imperative update to the previous literature review that only drew on 9 studies.^23^ Our search yielded 30 novel studies that were *excluded* from the previous review yet are relevant in providing insight into the use of haptics in vs These studies bolster this review and provide valuable insight to the field, especially as the previous review highlights a paucity of studies.^23^ While the previous review was beneficial at the time, this rapidly evolving field of research necessitates a more thorough summary of the literature, especially one that better conforms to expectations and standards for a review (eg, higher number of included studies). Towards this end, since 2019, 12 *additional* relevant studies have been published, over double the quantity of studies included in the previous review, further demonstrating the need for an update.

Our review introduces novel investigations that separates it from the previous study.^23^ While both reviews delve into study characteristics, populations, and tasks, our work stands out by encompassing a broader array of study designs, including crossover studies and quasi-experimental approaches, in addition to RCTs, providing a more comprehensive review of research methodologies. Moreover, our review delves into the characteristics of haptic feedback, examining aspects like collision and touch, drilling, and force changes during interactions with different tissue types. This detailed exploration enriches the understanding of haptic technology’s impact on learning and skill acquisition. Additionally, our review employs a more extensive range of methodological quality assessment tools, such as the MERSQI, CASP RCT standard checklist, and the JBI critical appraisal checklist for quasi-experimental studies. This rigorous evaluation enhances the reliability of our findings and emphasizes the strengths of our review in advancing the field.

Most studies utilized crossover and RCT methodologies to compare haptics and non-haptics. While RCTs are the gold standard for evaluation interventions, most studies had small samples sizes (as low as n = 8).^87^ This casts doubt on whether sufficient power for analyses existed and whether statistical assumptions were met.^88^ Indeed, working with highly specialized populations (eg, surgical residents, physicians) poses a barrier to conducting rigorous research with ample sample sizes; however, such a limitation ultimately impacts the validity of findings.^89^ Future work needs to strategically select research designs to bolster samples size whenever possible. For example, crossover methodologies can be utilized so that each participant provides two data points rather than one.^90^ This aids in achieving adequate power to detect statistical significance, providing higher quality results.^90^

Results also showed that most studies used oral instructions and hands-on practice to prepare students before the intervention, but nearly 25% didn’t provide any orientation. This lack of orientation can potentially skew results because unfamiliar participants might initially perform poorly but improve with practice, leading to potential misinterpretation of findings.^91^ This is especially important in our sample of studies as the most common research design was crossovers. Additionally, most studies relied on post-intervention assessments for comparisons, while some used pre/post designs, and only handful used longitudinal methods. Pre/post designs provide a more nuanced understanding of learning as they can factor in variance between groups and within participants.^92^ Future research comparing haptic and non-haptic approaches should combine verbal instructions and hands-on practice before assessment, and move beyond traditional post-intervention assessments. A recommendation to follow longitudinal designs may be unreasonable given the specialized study cohorts within surgical education. As such, future VS work should at the very least utilize pre/post assessment styles whenever possible.

Our review found that haptic technology’s application in surgical VS education reveals notable heterogeneity, influenced by the specific surgical domains it serves. In areas like dentistry and orthopedic surgery, the prevalent choice is to simulate drilling sensations, although the empirical support for their realism and effectiveness is limited. Furthermore, only four studies aimed to evaluate haptic feedback delivered through complex textures of various tissues, highlighting a gap within the literature. Interestingly, within laparoscopic simulations, the primary emphasis has centered on honing fundamental technical skills, such as instrument manipulation, grasping, and precise dissection in virtual environments. However, there exists an evident need for further advancements in replicating and delivering the nuanced tactile properties of soft tissues and solid organs, suggesting a promising avenue for continued research and development in this intersection of technology and surgical training for both educators and VS manufacturers.

Furthermore, our review showcases that haptic feedback has a generally positive impact on assessed tasks. Remarkably there were only three studies that demonstrated definitive drawbacks of haptic integration—though in two, the authors inferred this to be due to their device’s poor mechanics.^66,86^ The third study compared a printed temporal bone model with a virtual haptic simulator of a similar model.^59^ A printed model of bone is inherently more comparable to cadaveric bone, and hence better captures realism. However, the virtual haptic model was seen as having no differences in anatomic features, ease of use, ease of visual learning, and acquiring surgical skills relative to the printed model.^59^ This highlights the benefits of haptic integration within VSs as an adjunct to traditional teaching methodologies. It also demonstrates that when there is limited exposure to animal models and cadavers (eg, for novices and medical students), VSs with haptic feedback may be an invaluable alternative.^93^ Notably, we also found that many studies with non-significant results had means that often favored the haptic condition.

Given the limited sample sizes of included studies, perhaps many were unable to attain enough statistical power to detect significance.^94^ It is therefore plausible that any levels of medical learners could benefit from haptic feedback for their technical skill acquisition and maintenance. For residents, haptic feedback can be a tool to aid the early stages of skill acquisition while experienced physicians may use it to refine and enhance pre-existing skills, especially those in surgical specialties. Those in specialties where haptic cues are less critical may not benefit from haptic feedback to the same degree. This is because haptic feedback seems to benefit most in procedural training than training for other types of skills (eg, problem-solving, communication skills). Thus, surgical VSs should consider integrating haptic feedback whenever possible.

Next, few studies also showed mixed results where the haptic condition was favored in select assessed tasks but not others. Vamadevan and colleagues showcased that haptics led to accelerated learning but not the transferability of those skills to other non-haptic environments.^81^ Transferability of technical skill acquired during simulations into clinical practice is crucial.^95^ However, since the simulation (eg, in a haptic VS simulator) and real-life practice both serve as haptic conditions, these results are not necessarily against haptic-based VS training. Further work needs to be conducted to assess transferability of skills from virtual reality simulation to another real-life environment. Next, a study by Yovanoff and group demonstrated how haptic feedback can be a great tool for learning pressure or torque cues during training.^68^ However, they show that haptic feedback is not a one-size-fits-all solution for other aspects of surgical training such as procedural knowledge (eg, locating the needle on ultrasound).^68^ This provides support for haptic integration as a valuable educational tool for certain aspects of surgical training but not as a replacement for traditional training. Thus, it’s crucial to choose the appropriate training method considering the specific task being trained, as haptic feedback may not be universally effective for all task types. Interestingly, Yovanoff and colleagues also reported the frequency of feedback to be significantly higher using a simulator rather than a human instructor.^68^ This adds to the potential benefit of utilizing VS simulators as they can provide feedback more frequently, and in doing so, potentially alleviate human resources for more personalized teaching and learning. Importantly, the utilization of VSs for feedback should only be considered if the simulator has been adequately validated as frequent feedback does not necessarily mean good quality or accurate feedback.^96^ Lastly, LeBlanc and colleagues show how VS with haptic feedback offers significantly more objective results in standardized evaluations but is undesirable to residents due to its novel nature, partly explaining the longer task completion time.^52^ As such, we suggest progressive integration of haptic simulators starting with basic skill (eg, knot tying) at early stages of curricular training followed by progression to complex surgical tasks (eg, tumor resection in a haptic-enabled VS) at later stages to allow students to gain familiarity over time. Curricular integration should always be complemented with hands-on practice with the simulators prior to training sessions. By employing these guidelines, we can best tailor to the specific needs of learners and the procedures that they are involved in while optimizing the benefits of haptic technology.

### Limitation of Studies

In conducting this review, we rigorously assessed included studies. Many studies included in this review lost points in the MERSQI assessment due to a combination of small sample sizes and a lack of reporting on the validity evidence of their evaluation instruments.^32^ Most studies also lost points as they were single-centre studies. These limitations underscore the challenges inherent in conducting surgical education research, particularly when working with highly specialized populations. Additionally, the CASP RCT Standard Checklist and the JBI Critical Appraisal Checklist for Quasi-Experimental Studies observed that many studies fell short in achieving blinding of participants and researchers, a practical challenge often encountered in the context of medical training.^33,34^ The limitations in study quality, such as small sample sizes and challenges with blinding, should be considered when interpreting the results of this review. These limitations highlight the need for future research to address methodological shortcomings to enhance the robustness of findings in this field.

Next, the studies in this review are also subject to several inherent limitations. Firstly, the use of simulators across studies was extremely heterogeneous with varying companies, software versions, and levels of validation. This impacts interpretation of results across studies. For example, discrepancies in outcomes between studies employing the same simulator may arise from the use of different software versions. Failure to disclose such details can potentially lead to erroneous conclusions regarding the efficacy of a simulator (eg, deemed falsely ineffective due to outdated software). As such, future studies should explicitly note the version of the software they used. Furthermore, there is a need for standardization in the field to ensure interoperability and consistency in validation of VS hardware, requiring collaborations between researchers and VS manufacturers. Prioritizing partnerships with academic institutions and researchers will facilitate proper integration of haptic-enabled VSs, helping to ensure their alignment with proven educational outcomes. Continuous collaboration is crucial for tailoring simulator development to evolving training needs and ensuring a more effective and user-centric virtual surgical education experience. Moreover, these inconsistencies prevail in the types of methodologies adopted for training sessions and performance assessments. While the general study design may remain the same, nuances in how data is collected, and varying use of specific instruments makes direct comparisons across studies challenging. These also present challenges when attempting to draw generalizations. As such, conducting a meta-analysis of these studies was not feasible. The wide-ranging differences across these parameters preclude meaningful statistical synthesis and underscore the importance of a qualitative assessment approach in this comprehensive review.

## Conclusion

In conclusion, this updated review underscores the need for a fresh perspective on the application of haptic technology in surgical VSs. We highlight the rapid advancements made since the previous review and identify many studies not encompassed by the previous publication. Drawing on a significantly expanded pool of studies, we observed a notable heterogeneity in haptic utilization across surgical domains. Dentistry and orthopedic surgery have leaned toward simulating drilling sensations, although empirical support remains limited. Conversely, laparoscopic simulations primarily target fundamental technical skills but lack the replication of nuanced tactile properties in soft tissues and solid organs. Our findings generally highlight the positive impact of haptic feedback on assessed tasks, with only a few studies showing mixed and negative results. Importantly, these results do not undermine the value of haptic-based virtual surgical training but underscore its role as an adjunct to traditional teaching methodologies. Frequent haptic feedback in virtual simulators is advantageous for efficient and personalized learning, although careful consideration of simulator validation is essential. In summary, haptic integration in surgical education holds promise for enhancing training, emphasizing the need for continued research and development in this field.

## Supplemental Material

Supplemental Material - Findings Favor Haptics Feedback in Virtual Simulation Surgical Education: An Updated Systematic and Scoping Review Supplemental Material for Findings Favor Haptics Feedback in Virtual Simulation Surgical Education: An Updated Systematic and Scoping Review by Sayed Azher, Aralia Mills, Jinzhi He, Taliah Hyjazie, Junko Tokuno, Andrea Quaiattini, and Jason M. Harley in Surgical Innovation

Supplemental Material - Findings Favor Haptics Feedback in Virtual Simulation Surgical Education: An Updated Systematic and Scoping Review Supplemental Material for Findings Favor Haptics Feedback in Virtual Simulation Surgical Education: An Updated Systematic and Scoping Review by Sayed Azher, Aralia Mills, Jinzhi He, Taliah Hyjazie, Junko Tokuno, Andrea Quaiattini, and Jason M. Harley in Surgical Innovation
