# Supplementary material for: Findings Favor Haptics Feedback in Virtual Simulation Surgical Education: An Updated Systematic and Scoping Review
Source: Surg Innov. 2024 Mar 14;31(3):331–41. doi: 10.1177/15533506241238263 (PMC11047018; doi:10.1177/15533506241238263)
Supplement: Supplemental Material - Findings Favor Haptics Feedback in Virtual Simulation Surgical Education: An Updated Systematic and Scoping Review [file sj-pdf-1-sri-10.1177_15533506241238263.pdf]

Ovid MEDLINE Search Strategy

1 virtual reality/
2 (virtual realit* or vr).mp.
3 Simulation Training/ or High Fidelity Simulation Training/
4 (simulation* or simulator* or simulated or simulating).ti,ab,kf.
5 1 or 2 or 3 or 4
6 Haptic Technology/
7 (haptic or haptics or force feedback*).mp.
8 6 or 7
9 exp Specialties, Surgical/
10 exp Surgeons/
11 exp Surgical Procedures, Operative/
12 Surgery Department, Hospital/
13 su.fs.
14 (surger* or surgic* or surgeon*).ti,ab,kf.
15 (procedure* or operati*).ti,kf.
16 (procedure* or operati*).ab. /freq=2
17 (electrosurg* or microsurg* or neurosurg* or laparotom* or endoscop* or enteroscop* or laparoscop* or transplant* or retransplant* or pretransplant* or posttransplant* or shunt? or bypass or by-pass or biopsy or biopsies or graft* or ablation or ablate or ablated or debride* or debriding or amputate* or amputation* or anastomos*).ti,ab,kf.
18 (dissect or dissected or dissection* or microdissect* or excision or excise or resect*).ti,ab,kf.
19 9 or 10 or 11 or 12 or 13 or 14 or 15 or 16 or 17 or 18
20 5 and 8 and 19

Embase Classic + Embase Search Strategy

1 virtual reality/
2 (virtual realit* or vr).mp.
3 simulation training/ or high fidelity simulation training/
4 (simulation* or simulator* or simulated or simulating).ti,ab,kf.
5 1 or 2 or 3 or 4
6 haptic interface/
7 haptic technology/
8 tactile feedback/
9 (haptic or haptics or force feedback*).mp.
10 6 or 7 or 8 or 9
11 exp *surgery/
12 exp *surgeon/
13 su.fs.
14 (surger* or surgic* or surgeon*).ti,ab,kf.
15 (procedure* or operati*).ti,kf.
16 (procedure* or operati*).ab. /freq=2
17 (electrosurg* or microsurg* or neurosurg* or laparotom* or endoscop* or enteroscop* or laparoscop* or transplant* or retransplant* or pretransplant* or posttransplant* or shunt? or bypass or by-pass or biopsy or biopsies or graft* or ablation or ablate or ablated or debride* or debriding or amputate* or amputation* or anastomos*).ti,ab,kf.
18 (dissect or dissected or dissection* or microdissect* or excision or excise or resect*).ti,ab,kf.
19 11 or 12 or 13 or 14 or 15 or 16 or 17 or 18
20 5 and 10 and 19

Cochrane Central Register of Controlled Trials Search Strategy

#1 MeSH descriptor: [Virtual Reality] this term only
#2 (("virtual" NEXT realit*):ti,ab,kw OR vr:ti,ab,kw)
#3 [mh ^"Simulation Training"] OR [mh ^"High Fidelity Simulation Training"]
#4 (simulation*:ti,ab,kw OR simulator*:ti,ab,kw OR simulated:ti,ab,kw OR simulating:ti,ab,kw)
#5 #1 OR #2 OR #3 or #4
#6 [mh ^"Haptic Technology"]
#7 (haptic:ti,ab,kw OR haptics:ti,ab,kw OR ("force" NEXT feedback*):ti,ab,kw)
#8 #6 OR #7
#9 [mh "Specialties, Surgical"]
#10 [mh Surgeons]
#11 [mh "Surgical Procedures, Operative"]
#12 [mh ^"Surgery Department, Hospital"]
#13 (surger*:ti,ab,kw OR surgic*:ti,ab,kw OR surgeon*:ti,ab,kw)
#14 (procedure*:ti OR operati*:ti,ab,kw)
#15 (electrosurg*:ti,ab,kw OR microsurg*:ti,ab,kw OR neurosurg*:ti,ab,kw OR laparotom*:ti,ab,kw OR endoscop*:ti,ab,kw OR enteroscop*:ti,ab,kw OR laparoscop*:ti,ab,kw OR transplant*:ti,ab,kw OR retransplant*:ti,ab,kw OR pretransplant*:ti,ab,kw OR posttransplant*:ti,ab,kw OR shunt?:ti,ab,kw OR bypass:ti,ab,kw OR by-pass:ti,ab,kw OR biopsy:ti,ab,kw OR biopsies:ti,ab,kw OR graft*:ti,ab,kw OR ablation:ti,ab,kw OR ablate:ti,ab,kw OR ablated:ti,ab,kw OR debride*:ti,ab,kw OR debriding:ti,ab,kw OR amputate*:ti,ab,kw OR amputation*:ti,ab,kw OR anastomos*:ti,ab,kw)
#16 (dissect:ti,ab,kw OR dissected:ti,ab,kw OR dissection*:ti,ab,kw OR microdissect*:ti,ab,kw OR excision:ti,ab,kw OR excise:ti,ab,kw OR resect*:ti,ab,kw)
#17 #9 OR #10 OR #11 OR #12 OR #13 OR #14 or #15 OR #16
#18 #5 AND #8 AND #17

Expanded Version of Table 2

Table 2.

Assessed Parameters, Including Objective (time, path length, movements, etc.) and Subjective (ease of use, preference, etc.) Tasks

Tasks being Assessed	N
**Surgical Tasks**	101
Time Measurement	20
Time-to-task completion time	10
Operating time	1
Time spent in training course	1
Time to reach predefined proficiency	3
Instructor time spent on feedback during training	1
Average speed of each hand (cm/s)	1
Speed of usage of surgical instruments	1
Best training trial speed	1
Variance in task completion time across each session	1
Subjective Assessment	17
Usefulness of the virtual haptic platform	5
Experience[††]	6
Ease of use	1
Perception of graphical aspects of virtual platform	2
Post-trial self-ratings of performance	1
Self-efficacy	1
Surgical patients' level of discomfort	1
Objective Assessment	32
Video assessment of performance[†]	4
Clinical testing assessment	2
Training effectiveness	3
Anatomy comprehension improvement after training	1
Written examination improvement after training	1

Fluoroscopy image evaluation	1
Attempts to reach proficiency	1
Number of trials needed to reach proficiency	4
Average performance pre and post training course	1
Learning curve analysis	1
Written assessment*	3
Learning curve analysis	1
Quality of surgical procedure	1
Lateral fluoroscopic image evaluation	1
Procedure completed (%)	1
Amount of instructor assistance on procedure	1
Number of tool insertion attempts	1
Number of unsuccessful tool insertion attempts	1
Number of pictures during task with respect to camera changes	1
Average moving distance of surgical instrument	1
Average error length of surgical instrument	1
<u>Instrument Dexterity</u>	27
Total length of the tool tip trajectory	1
Number of tool-target collisions	1
Distance of penetration into the target	2
Amount of force applied on the target	1
Maximum distraction of the fraction between groups	1
Amounts of angulation in virtual tool	1
Maximum stretch damage to surgical tissue	2
Maximum damage to target (mm)	1
Number of damages to the surgical tissue	1
Number of errors made during testing	3
Angular path length between target and simulator tool	2
Total tissue damage	1

Instrument path length	3
Right and left hand error comparison	1
Amount of grasping tension of surgical tool	1
Economy of movement of each hand (%)	1
Instrument navigation accuracy	2
The distance between the optimal and actual entry point at the styloid radial process	1
Mandibular fracture reduction	1
Orthopedic Specific Tasks	2
The distance between the optimal and actual entry point at the styloid radial process	1
Mandibular fracture reduction	1
Medical Scanning**	3
Amount of simulated tumor volume within the boundaries of the reference volume (PercentageIN)	1
Percentage of simulated tumor volume falling within the reference tumor volume (TotalScanned)	1
Difference in scanning between haptic modalities (BinaryCount)	1
**Dental Specific Tasks**	31
Cavity inspection	10
Mean time of the cavity preparation after haptics training	1
Pulpal floor smoothness	1
Pulpal floor direction	1
Buccal wall direction	1
Lingual wall direction	1
Mesial wall direction	1
Mesial wall smoothness	1
Distal wall smoothness	1
Internal line angle	1
Internal point angle	1
Implantology-related drilling outcomes	12
Mean position time	1

Mean angulation deviation	1
Mean drilling depth	1
Perforation (%) per trial	1
Mean drilling duration	1
Mean total duration	1
Mean buccolingual angle deviation	1
Mean mesiodistal angle deviation	1
Mean drilling depth	1
Mean position difference	1
Mean total duration of drilling	1
Site preparation (%) without perforation	1
Molar preparation	3
Evaluation by experts of precision of molar preparation	2
Ease of use of the virtual platform rated by participants	1
Molar insertion task	6
Time-to-task completion	1
Amount of assistance given my instructor	1
Usefulness of virtual training platform	1
Impact of psychomotor skills on confidence	1
Realism of graphical images displaced	1
Realism of the tactile sensations of the haptic device	1
**Non-Surgical Tasks**[‡]	12
Time Measurement	1
Time-to-task completion time	1
Subjective Assessment	9
Pain intensity	2
Virtual image quality rating	2
Preference for haptic vs non-haptic environment	3
Mental effort	1
Ease of use	1

Objective Assessment	2
Performance after training	1
Change of performance over time	1

Notes.

†Video assessment of performance was based on objective structured assessment of technical skills (OSATS) criterion, rated by experts[1]

††Subjective reporting of experience included subjective experience with the feedback category[2–4]; decision making process[2]; experience with haptic vs non haptic environment[5–8]; experience in anatomical features[9], learning cortical mastoidectomy[10]; experience learning a posterior tympanotomy/facial recess approach to the middle ear[10]; and participants' experience using a new virtual laparoscopic platform.[8,11]

*Written test based on three anatomy tests consisting of eight questions with questions 1-3 being identification questions and 4-8 being multiple choice.[12] Question 1 had nine elements, question 2 had six elements, and question 3 had ten elements.[12]

**Two specific metrics were used for the comparison of generated and reference volumes: percentageIN, which represented the amount of simulated tumour volume with the boundaries of the reference tumour volume, and totalscanned, representing the percentage of simulated tumour volume falling within the reference tumour volume boundaries.[2] To assess overall performance, a binary variable called binary count was utilized.[2] This variable measured the difference in PercentageIN, referred to as PercentageINdifference, between scanning with a specific feedback modality and scanning without any feedback for a given data set.[2]

‡Non-surgical tasks included synthetic cylinder volume task[3], moving intact vs virtually affected hand[13], fine dissection virtual task[14], diathermy task[15], cognitive load test[16], ultrasound simulator task.[17]

References

1. Balci C, Tas T, Hazar A, et al. Applicability and effectiveness of virtual reality simulator training in urologic surgery: A double-blind randomised study. *Nobel Medicus*. 2014;10:66-71.
2. Camara M, Mayer E, Darzi A, Pratt P. Intraoperative ultrasound for improved 3D tumour reconstruction in robot-assisted surgery: An evaluation of feedback modalities. *Int J Med Robot*. 2019;15(2):e1973. doi:10.1002/rcs.1973
3. Deng S, Singh E, Wheeler G, et al. P1566 Evaluation of haptic feedback for interaction with volumetric image data in virtual reality. *European Heart Journal - Cardiovascular Imaging*. 2020;21(Supplement_1):jez319.986. doi:10.1093/ehjci/jez319.986
4. Chen HE, Sonntag CC, Mirkin KA, et al. From the simulation center to the bedside: Validating the efficacy of a dynamic haptic robotic trainer in internal jugular central venous catheter placement. *Am J Surg*. 2020;219(2):379-384. doi:10.1016/j.amjsurg.2019.10.026
5. Hedman L, Ström P, Andersson P, Kjellin A, Wredmark T, Felländer-Tsai L. High-level visual-spatial ability for novices correlates with performance in a visual-spatial complex surgical simulator task. *Surg Endosc*. 2006;20(8):1275-1280. doi:10.1007/s00464-005-0036-6
6. Chmarra MK, Dankelman J, van den Dobbelsteen JJ, Jansen FW. Force feedback and basic laparoscopic skills. *Surg Endosc*. 2008;22(10):2140-2148. doi:10.1007/s00464-008-9937-5
7. Zhou M, Tse S, Derevianko A, Jones DB, Schwaitzberg SD, Cao CGL. Effect of haptic feedback in laparoscopic surgery skill acquisition. *Surg Endosc*. 2012;26(4):1128-1134. doi:10.1007/s00464-011-2011-8
8. Dwisaptarini AP, Suebnukarn S, Rhienmora P, Haddawy P, Koontongkaew S. Effectiveness of the Multilayered Caries Model and Visuo-tactile Virtual Reality Simulator for Minimally Invasive Caries Removal: A Randomized Controlled Trial. *Oper Dent*. 2018;43(3):E110-E118. doi:10.2341/17-083-C
9. Benjamin MW, Sabri O. Using Haptic Feedback in a Virtual Reality Bone Drilling Simulation to Reduce Plunge Distance. *Cureus*. 13(9):e18315. doi:10.7759/cureus.18315
10. Hochman JB, Rhodes C, Kraut J, Pisa J, Unger B. End User Comparison of Anatomically Matched 3-Dimensional Printed and Virtual Haptic Temporal Bone Simulation: A Pilot Study. *Otolaryngol Head Neck Surg*. 2015;153(2):263-268. doi:10.1177/0194599815586756
11. Hogle NJ, Widmann WD, Ude AO, Hardy MA, Fowler DL. Does training novices to criteria and does rapid acquisition of skills on laparoscopic simulators have predictive validity or are we just playing video games? *J Surg Educ*. 2008;65(6):431-435. doi:10.1016/j.jsurg.2008.05.008
12. Erolin C, Lamb C, Soames R, Wilkinson C. Does Virtual Haptic Dissection Improve Student Learning? A Multi-Year Comparative Study. *Stud Health Technol Inform*. 2016;220:110-117.
13. Sano Y, Wake N, Ichinose A, et al. Tactile feedback for relief of deafferentation pain using virtual reality system: a pilot study. *J Neuroeng Rehabil*. 2016;13(1):61. doi:10.1186/s12984-016-0161-6
14. Våpenstad C, Hofstad EF, Bø LE, et al. Limitations of haptic feedback devices on construct validity of the LapSim® virtual reality simulator. *Surg Endosc*. 2013;27(4):1386-1396. doi:10.1007/s00464-012-2621-9
15. Ström P, Hedman L, Särnå L, Kjellin A, Wredmark T, Felländer-Tsai L. Early exposure to haptic feedback enhances performance in surgical simulator training: a prospective randomized crossover study in surgical residents. *Surg Endosc*. 2006;20(9):1383-1388. doi:10.1007/s00464-005-0545-3
16. Cao CGL, Zhou M, Jones DB, Schwaitzberg SD. Can Surgeons Think and Operate with Haptics at the Same Time? *J Gastrointest Surg*. 2007;11(11):1564-1569. doi:10.1007/s11605-007-0279-8
17. Chao C, Chalouhi GE, Bouhanna P, Ville Y, Dommergues M. Randomized Clinical Trial of Virtual Reality Simulation Training for Transvaginal Gynecologic Ultrasound Skills. *J Ultrasound Med*. 2015;34(9):1663-1667. doi:10.7863/ultra.15.14.09063
