# Supplementary material for: Findings Favor Haptics Feedback in Virtual Simulation Surgical Education: An Updated Systematic and Scoping Review
Source: Surg Innov. 2024 Mar 14;31(3):331–41. doi: 10.1177/15533506241238263 (PMC11047018; doi:10.1177/15533506241238263)
Supplement: Supplemental Material - Findings Favor Haptics Feedback in Virtual Simulation Surgical Education: An Updated Systematic and Scoping Review [file sj-pdf-2-sri-10.1177_15533506241238263.pdf]

Appendix

Figure 1.

PRISMA flow diagram

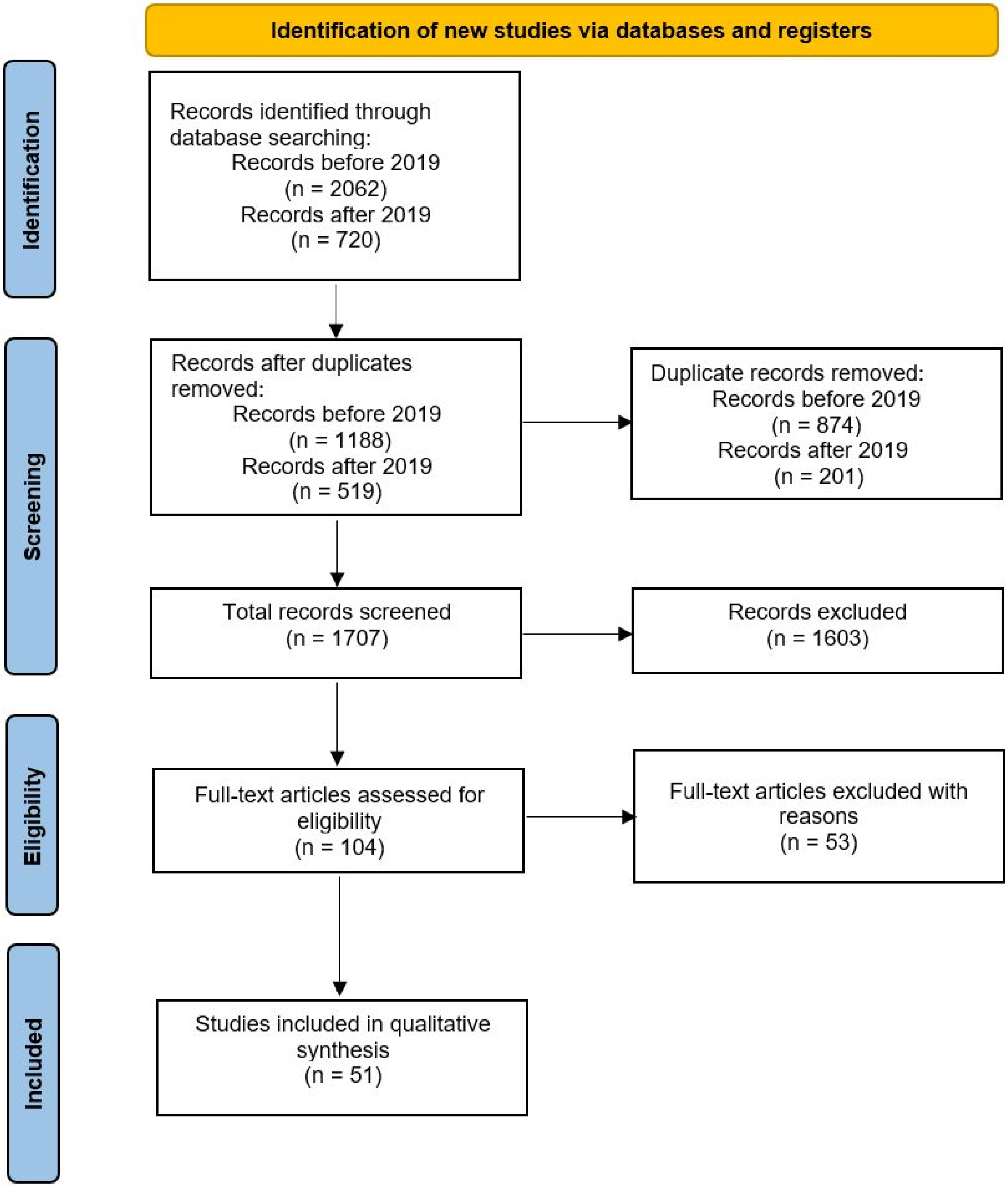

Table 1.

Study Demographics and Outcomes

Author	Year	Country	Study Type	n	Participant Type	Simulation Device	Comparison	Training Task(s)	Assessed Task	Outcome Assessment	Outcome Data	Strength of Findings
Kim	2004	USA	Experimental study	24	Novices	PHANTOM 1.5 Device	H v NH	Detail description of tasks and scoring	Bimanual pushing and cutting simulating Heller's myotomy on non-VR simulator	Data on completion time, instrument path length, right and left-hand errors, grasping tension	For the more difficult cutting, the time to complete the tasks was shorter with haptics, at all levels of difficulty ($p < 0.05$). Grasping tension was shorter at level 1 (11.2 ± 3.3 versus 7.9 ± 1.8). Data on instrument path length and errors trended towards improve efficiency from haptics at all difficulty levels but did not achieve statistical significance.	+/-
Gerovich	2004	USA	Randomized crossover study	10	Novices	Impulse Engine®	H (Visual cues differed) v NH (Visual cues differed) v Limited Visual Feedback (Haptics differed)	Familiarization with Impulse Engine haptic interface and different needle insertion scenarios	Needle insertion into multilayer tissue with varying haptic and visual cues	Error between penetration depth and layer boundary for skin, fat, and muscle in each test	Performance with haptics was the same regardless of prior needle insertion experience. Haptics reduced error in both groups for all layers improving layer transition detect by at least 52%. Static display of tissue layers was not necessary if haptics was provided indicating that visibility of tissue boundaries has little effect on performance.	+
Gosling	2005	Germany	Randomized crossover study	1	Orthopedic Physician	Custom Robot (RX 60)	H v Manual	Finished learning curve for each technique	Reduction of femur	Maximum distraction, number of pictures with respect to camera changes, reduction time, amount of angulation, translation after reduction	Haptics reduced the time taken for manual reduction procedure ($p < 0.0001$). Haptics in the manual reduction group led to better outcomes in terms of varus/valgus deformity ($p = 0.034$). Haptics in the manual reduction group resulted in reduced ante-/recurvature deformity ($p < 0.01$). Haptics did not contribute significantly to reducing malrotation in the manual reduction group ($p < 0.001$). Haptics in the robot-assisted reduction group resulted in improved image control, requiring fewer camera changes. Haptics in the robot-assisted reduction group led to reduced distraction within the fracture gap ($p < 0.001$).	+
Ström	2006	Sweden	Randomized crossover study	38	Medical Residents	Procedicus Abdomen	H v NH	Psychometric tests (mental rotation, cognitive ability) before haptic training; diathermy tasks	Two diathermy task, both on medium difficulty	Flow and Borg CR10 questionnaires; simulator tasks (manipulate diathermy (MD), point diathermy (PD))	No significant difference in visual spatial ability (BASIQ and MRT-A tests). Flow questionnaire and Borg CR10 showed no significant differences. After 1 hour of training, no significant differences in MD and PD tasks. After 2 hours of training, haptics showed better performance in both MD and PD tasks ($p = 0.01$, $p < 0.05$). Only haptics-first group showed improvement during the second session in both MD and PD tasks ($p < 0.001$ and < 0.01).	+
Cohen	2006	USA	RCT	45	Physicians	GI Mentor	H v NH	General lectures on colonoscopy; group A received simulator training on the GI Mentor simulator; warm-up exercises and specific simulated activities	Colonoscopies	Perspectives questionnaire on usefulness of training; total procedure time, time to reach the cecum, percentage of mucosal surface examined, number of episodes of excessive pressure; colonoscopy competency form	Haptics training rated as moderately useful to useful. Mixed effects model showed that the haptics group performed better overall in terms of objective competence ($p < 0.001$) of which the difference was larger in earlier blocks. There was no difference in the number of blocks needed for fellows to reach proficiency in the haptics vs non-haptics groups.	+

Hedman	2006	Sweden	Randomized crossover study	54	Medical Students	Procedicus KSA + Procedicus MIST	H v NH	Instrument navigation task training in Procedicus KSA with anatomic graphics and haptics (haptic); manipulate and diathermy task training in Procedicus MIST with no anatomic graphics and no haptics (non-haptics)	Procedicus KSA task evaluated on navigation of optic device and probe to probe spheres in the virtual upper abdomen; Procedicus MIST task evaluated performance in manipulate and diathermy task	Psychometric test; performance in simulator tasks assessing time, movement economy, collisions, total score; correlations with psychometric test score	Virtual haptic-simulated repair did not significantly differ from surgical repair ($p = 0.42$). Virtual CAD-simulated repair (non-haptics) differed from surgical repair ($p = 0.02$). Mean measurements between haptic- and CAD- (non-haptics) differed ($p = 0.006$). Surgical values were greater than haptics ($p = 0.03$). More consistency between users for haptics than CAD- ($p \leq 0.001$). Self-reported quality of repair and intuitiveness was higher for haptics than CAD ($p = 0.04$ and $p = 0.003$). No difference in the number of views used between CAD and haptics ($p = 0.6$). There was a significant improvement after 1 h of training in the Procedicus MIST regarding total score ($p < 0.001$) and in the Procedicus KSA regarding time, movement economy, collisions with the probe, and total score ($p < 0.001$). High-level visual-spatial ability is important for surgical novices to possess in the early training phase	+
Cao	2007	USA	Crossover study	30	Medical Residents	MIST-VR + ProMIS Systems	H v NH + Cognitive Load (Loaded v Unloaded)	Demonstration of task and oral instruction; practice until one target drop error-free trial was achieved on each simulator	Transfer-place task using MIST-VR and ProMIS systems; Cognitive load (mental arithmetic); performance of real colonoscopies after the training period	Time-to-task completion, number of errors, total number of math problems completed	Faster task completion with haptic feedback ($p < 0.001$)<br>Cognitive load had a larger impact on completion time without haptics ($p < .001$). Haptics had fewer errors ($p < 0.001$). Slight positive correlation between time-to-task completion and number of math problems in both haptics ($r = 0.24$) and no-haptics ($r = 0.26$) conditions.	+
Chmarra	2008	Netherlands	Randomized crossover study	19	Medical Residents (Gynecology)	SIMENDO VR Trainer	H v NH	None	Box tasks including balls, ring, elastic band tasks	Demographics questionnaire, prior experience in laparoscopic procedures questionnaire, performance evaluated based on time, path length, depth perception	No significant difference in laparoscopic surgery experience between groups (Box = haptic; VR = non-haptic). No significant differences in time, path length, or depth perception for balls and ring tasks in both trainers. Box-VR group performed 50% faster than VR-box ($p < 0.01$) in the elastic band task. Box-VR group had shorter path length for left (60%, $p < 0.01$) and right (55%, $p < 0.01$) instrument. Box-VR group had shorter depth perception for left (65%, $p < 0.01$) and right (50%, $p < 0.01$) instruments. VR trainer showed significant difference only for right instrument: path length 70% shorter ($p < 0.01$), depth perception 65% shorter ($p < 0.02$).	+/-
Hogle	2008	USA	RCT	21	Medical Residents	LapSim®	H v NH	Lecture on laparoscopic cholecystectomy; LapSim Basic Skills curriculum	Laparoscopic cholecystectomies in pigs	GOALS tool evaluation by skilled laparoscopic surgeons (tracked clinical performance on laparoscopic cholecystectomies in haptics and non-haptics groups) – evaluated on camera navigation, instrument navigation, coordination, grasping, lifting and grasping, cutting, and clip applying; learning rate according to LapSim simulator (haptics)	Haptics showed improvement in depth perception in the operative performance of cholecystectomies ($p = 0.04$). No significant results for the untrained (non-haptics) group ($p = 0.29$). Non-significant improvement in bimanual dexterity, efficacy, tissue handling, and autonomy in the haptics group.	+
Panait	2009	USA	Crossover trial	10	Medical Students	Laparoscopy VR System	H v NH	Familiarization with device and tasks	Drills on laparoscopic VR at 3 levels of difficulty with a 3-hour gap between haptics and non-haptic	Completion time, instrument path length, right and left-hand errors, grasping tension	The peg transfer drill showed no difference in performance between the haptic and non-haptic simulations for all metrics at all three levels of difficulty. Task completion time, instrument path length, and error quantification demonstrated similar values in the haptic and non- haptic environments at all levels of difficulty. In more difficult cutting, time was shorter with haptics, at all levels of difficulty ($p < 0.05$). Grasping tension was shorter at level 1.<br>Instrument path length and errors trended towards	+/-

											improved efficiency from haptics at all difficulty levels but did not achieve statistical significance.	
Salkini	2010	USA	RCT	20	Medical Students	Simbionix Lap Mentor	H v NH	3 training tasks – task 1: Grasping and closing leaking tubes, task 2: Gently grabbing and cutting jelly plate attachments, task 3: Grasping and cutting gallbladder attachments	Task 1: Closing leaking tubes using duck grasp and clip applier. Task 2: Grasped jelly plate and cut attachments free. Task 3: Manipulate gallbladder, cutting its attachment via electrocautery	Economy of movement of each hand, average speed of each hand	No significant difference between haptics vs non haptics in accuracy, economy of movement, and speed of the non-dominant hand. Dominant hand's speed had a significant difference, with the haptics group showing slower movement compared to non-haptics group.	+/-
Thompson	2011	USA	RCT	33	Undergraduate, Medical, and Graduate Students	Simbionix Lap Mentor II	H v NH v C	Presentation on laparoscopic surgery and LapMentor II; practiced basic tasks until proficiency reached with and without haptics; all groups practiced procedural tasks and 10 laparoscopic cholecystectomies	VR laparoscopic cholecystectomies; 4 procedural tasks of cholecystectomies	Total time, efficiency of cautery, number of movements, total path length, speed of instruments; comparison of number of trials needed to reach proficiency; comparison of haptics vs non-haptics in cholecystectomy trials; learning curves for all parameters	Mixed results exist when using haptic-enabled LapMentor II VR laparoscopic training. Efficiency of cautery, speed of instruments, and number of trials needed to reach proficiency favoured haptics. Performance of haptic versus non-haptic trained participants in cholecystectomy trials had mixed results: There was a negative effect of haptics, increasing the number of right instrument movements (estimate of effect, 29; $p = 0.008$) while having a positive effect on the speed of the right instrument (estimate of effect, 0.2 cm/s; $p = 0.0004$). However, engaged haptics also improved (decreased) the number of left instrument movements (estimate of effect, −14; $p = 0.02$). Haptics training did not appear to show a benefit over non-haptics training with regard to improved learning	+/-
Zhou	2012	USA	Controlled trial	20	Undergraduate and Graduate Novices in Laparoscopy	MISTVR + ProMIS	H v NH	Brief introduction to laparoscopic surgery; demonstration of both open and laparoscopic suturing by an expert surgeon; 1-hour training for 6 days/week where they attempted as many knots as possible	Laparoscopic knot-tying task involving two single-loop, half square knots forming a complete square knot using two needle drivers; in non-haptics they sutured virtual organ on MIST-VR system; in haptics they sutured a Penrose drain fastened with Velcro to a block on the ProMIS system	Individual learning curved from time-to-task-completion measure; ANOVA to examine effect of training session; ANOVA to compare learning rates; paired t-test to compare time-to-task completion, variance of task completion time per session; and the best performance time of the session for the two groups	Non-haptics reached performance plateau by the 6th session, while the haptics group reached it earlier, by the 5th session. The overall learning rate comparison between haptics and non-haptics showed no statistical significance ($p < 0.061$). Haptics had slightly higher learning rate compared to non-haptics indicating a flatter learning curve and fewer trials needed to reach plateau. Individual learning curves showed a faster time to task completion in the haptics condition across the training session ($p < 0.04$). Haptics and non-haptics performed equally for every knot completed in training ($p < 0.653$). The best trial in each session for the haptics group was faster (0.006).	+
Lyu	2013	Taiwan	Crossover trial	8	Novices	PHANTOM Device	H v NH	Familiarization routine	Inspection with force guidance, a gap of 3 days followed by inspection with force guidance.	Operating time, normal path error	Performance with haptics was better than without – average error with force guidance is 33.01% lower and operating time is 14.95% less than without force guidance. Normal path error trended towards a benefit from the haptics in the inspection.	-
Sengül	2013	Switzerland	Randomized crossover study	20	Novices	Da Vinci Simulator	H v NH v Static	Oral instruction	Tool-use task followed by crossmodal congruency task	Mean reaction times, error rates per condition for each participant, congruency contingent effect	Haptics with force feedback increased the congruency contingent effect compared to the static condition ($p < 0.05$) and without-force feedback condition ($p < 0.05$). No significant difference in congruency contingent effect between the static condition and without-force feedback condition ($p = 0.59$).	-
Vapenstad	2013	Norway	Crossover trial	20	Physicians (Surgeons)	Xitact IHP + Xitact ITP	H v NH	None	Fine dissection, lifting and grasping	Questionnaire regarding perceptions of haptics and non-haptics	79% of surgeons found handles with haptic feedback important if they felt realistic. 70% of	+/-

											surgeons thought handles without haptic feedback felt most realistic, while 20% believed haptic handles imitated reality best. Of those who identified the haptic handle, 12% believed it provided haptic feedback successfully. 95% perceived high friction with haptic handles, while 5% perceived it as realistic. Regarding handles without haptic feedback, 45% perceived realistic friction, 50% too low, and 5% too high. 85% of participants performed best with handles without haptic feedback, 10% with haptic handles, and 5% noticed no difference (P < 0.001).	
Kulscar	2013	Ireland	Prospective interventional study	27	Medical Students	SenseGraphic Immerse Workbench	H v NH	Standardized tutorial via PowerPoint on spinal anesthesia; video by expert anesthesiologist performing spinal anesthesia; practical training session with an orange (non-haptics) or simulator (haptics)	Spinal anesthesia in the virtual and clinical environments	Objective structured assessment of technical skills (OSATS) by study-blinded expert; assessment of video films using OSATS scoring system; feedback questionnaire	No difference between haptics and non-haptics in written test. No difference on the global rating skills during simulator-based training. No statistical significance but greater trend towards greater scores in haptics (p = 0.06). Haptics scored greater than non-haptics after clinical testing assessment (p = 0.02). Both groups scored similarly on the video assessment of clinical performance. 28.5% of participants from the haptics group found the clinical procedure to be stressful compared to 75% in the non-haptics group. All respondents found learning on the simulator to be beneficial to practice before helping real patients.	+
LeBlanc	2013	Canada	Stratified Randomized crossover trial	27	Medical Residents (Orthopaedic Surgery)	Sawbones Simulator	H v NH	Brief training session; brief familiarization period	Internal fixation of the ulna with the use of a neutralization plate	15-task itemized check list; global rating scale form; time to complete procedure; simulator-specific questionnaire filled out by participants after the completion of the procedure	Residents performed better on the virtual haptic simulator in both the checklist score (p < 0.05) and global rating scale score (p < 0.05). However, the haptic group took about a minute longer to finish the procedure (p < 0.05). Residents found that the virtual simulator needed further improvement (p < 0.05) and that they would prefer the sawbones (non-haptic) simulator if given the choice (p < 0.05).	+
Liu	2013	China	RCT	18	Medical Students	TB Simulator	H v NH	Didactic lecture and PowerPoint on anatomy and dissection procedure; simulation group practiced on the simulator	Anatomy and dissection of temporal bone	Anatomy comprehension, magnitude of improvement from days 0 to 14	Both haptics and non-haptics groups showed improvement to comprehension of temporal bone anatomy. There was a 10% improvement in the non-haptics group as opposed to a 20% improvement in the haptics group (p < 0.05). Haptics group has a greater magnitude of improvement in the written examination (p < 0.05).	+
Balci	2014	Turkey	Double-blind randomized study	16	Physicians (Urologists)	LapSim®	H v NH	Practical and theoretical laparoscopic training course; group A VR simulator training system with haptics, group B conventional physical laparoscopic training box	Transperitoneal Laparoscopic Renal Cyst Decortication (TLRCD)	Video recordings, objective Structured Assessment of Technical Skills (OSATS) scale scores	Mean duration of operations in Groups A and B had no statistically significant difference between the groups (p = 0.72). All surgeons in both groups were successful based on OSATS assessment criteria, with no significant difference observed between the groups in terms of various assessment criteria, including respect to tissue, duration of operation and manipulations, instrumental experience, safety of manipulations, use of an assistant, flow of the operation, and accuracy of the operative technique (all p-values > 0.05).	+/-
Joseph	2014	France	RCT	60	Dentists + Dentistry Students	Virteasy Dental Simulator	H v NH v Experts	Instructional PowerPoint presentation; familiarization with simulator operations; detailed explanation on implant selection and scanning software and the aspects of implant	Drilling on resin model (implantology)	Evolution of drilling outcomes – mean position time, mean angulation deviation, mean drilling depth, perforation per trial, mean drilling duration, mean total duration	Evolution of drilling outcomes with simulation training (haptics): Improvement in mean position deviation between the 1st and 4th trial (p = 0.04) improved accuracy in the 8th trial compared to the first (p = 0.0288), improvement in drilling depth over time (p = 0.0310).	+

								planning such as diameter, implant shape, and positioning)				
Bouhelal	2014	UK	Experimental study w/comparative design	42	Novices	VR Simulator	H v NH	Unspecified	VR simulator with haptic vs without haptics (no details provided)	Mean total simulator time (MTST)	Proficiency in Basic tasks 5 in the Haptic was achieved in MTST of 12:49 in 7.3 trials compared to 16:28 minute in 7.7 trials for non-haptic. Proficiency in Basic tasks 6 in the Haptic was achieved in MTST of 12:20 min in 7.2 trials compared to 19:22 minute in 7.2 trials for non-haptic. Proficiency in Procedural tasks 3 in the Haptic was achieved in MTST of 26:42 in 5.3 compared to 59:19 minute in 12.4 for non-haptic. Proficiency in procedural tasks 4 in the Haptic was achieved in MTST of 27:40 in 5.2 trials compared to 1:05:25 minute in 8 trials for non-haptic. In full LC proficiency was achieved in MTST of 30:04 minute in 3.4 trials compared to 1:27:43 minute in 8.1 trials for non-haptic.	+
Hochman	2015	Canada	Controlled trial	10	Medical Residents	Voxel Based Model (VM)	H v NH	None	Drilling of identical virtual model, physical bone model, and cadaveric specimen	Experience survey comparing virtual model and physical bone model to cadaveric simulation where they rated physical characteristics, specific anatomic feature representation, usefulness of surgical skills training, and perceived educational value	Participants found several mechanical properties of the physical bone model (non-haptics) to be more comparable to a cadaveric bone than the virtual model (haptics) - differences in cortical ($p = 0.011$) and trabecular ($p = 0.004$) osseous realism, vibrational properties ($p = 0.001$), and air system generation ($p = 0.003$). No differences found between virtual and physical bone model in anatomic features. Both virtual and physical bone model found to be productive resources for acquiring surgical skills. Physical bone model ranked more effective for learning cortical mastoidectomy ($p = 0.015$) and the posterior tympanotomy/facial recess approach to the middle ear ($p = 0.023$). Physical bone model considered the superior tool in 7/9 domains ($p < 0.05$). No difference in ease of use and use as a visual learning tool.	+
Koo	2015	USA	RCT	34	Dentistry Students	Manual Dexterity Haptic Interface	H v NH	Didactic instruction; familiarization with haptic device and VR manual dexterity training with haptic device	Class II amalgam preparation in a mandibular first molar and Class III resin preparation in a maxillary first incisor using plastic typodont model as baseline	Evaluation form for tooth preparations; questionnaire for the experimental group to assess subjective evaluation of haptics exercise in preclinical training; post-haptic exercise survey for the experimental group	Haptics scored better in treatment of adjacent tooth ($p < 0.05$). 65% agreed that game-like features of software made experience more fun and interesting. Control and haptics agreed that haptics was easy to use, and that the device would be useful to have at home. 59% disagreed or strongly disagreed that the amount of time engaged with haptics was sufficient to evaluate effectiveness. 67% disagreed or strongly disagreed that it was easier to perform the task after the simulation exercises or that haptics improved manual dexterity for handling the handpiece.	+
Park	2015	USA	Crossover study	1	Physician (Surgeon)	MAKO Robotic-arm	H v NH	None	Manual resection of identical sawbone models of the proximal femur with a cam-type impingement deformity by a single surgeon; half was resected using an open free hand technique (non-haptics) and the other half underwent cam decompression with a robotic	Arc resection, volume of bone removed, resection depths; comparison with pre-operatively planned desired resection to determine resection error	Mean arc of resection error of the non-haptics group was higher than the haptics group ($p < 0.0001$). Haptics had lower start and finish mean error than the non-haptics group ($p < 0.0001$). Haptics resulted in less over-resection (mean volume error ($p < 0.01$). Both techniques showed similar mean maximum resection depth. The mean cutting time was shorter in the haptics group ($p < 0.001$).	+

									assisted technique from the MAKO RIO technology (haptics)			
Chao	2015	France	Randomized Clinical trial	44	Medical Residents	ScanTrainer Ultrasound Simulator	H v NH	VR simulation training using haptic transvaginal high-fidelity simulator; oral presentation with PowerPoint slides; videos; question period; instructed on how to operate main settings of ultrasound machine	Produce images (longitudinal and axial sections of the uterus and ovaries) and measure the uterus and each ovary meeting quality criteria using the ScanTrainer simulator on a new virtual patient with normal ovaries and a retroverted but otherwise normal 3D uterus	Image quality score calculated from a set of 4 images according to Salomon et al., rate of adequate images per group, questionnaire assessing perspectives on haptics vs non-haptics	Mean quality image score was higher in the haptics group ($p = 0.0302$). Rate of satisfactory images was higher in the haptics group, but not statistically significant. Confidence in scanning real patients improved in both groups by 100%. Perception of increased scanning speed was higher in the non-haptics group (94%) when compared to the simulation group (86%). Perception of capacity to produce adequate images was 100% in both groups. Perception of capacity to get oriented was 100% in both groups. All trainees agreed that non-haptics teaching remained useful in addition to haptics training.	+
Erolin	2016	UK	Longitudinal controlled trial	79	Undergraduate Anatomy Students + Graduate Anatomy Students	Touch X	H v Keyboard + Mouse v C	None	Cadaver dissection of hand/wrist; anatomy test	Anatomy test	Phase 1: Question 2 (identification question) was score significantly higher for haptic than control ($p = 0.03$). Phase 2: Question 2 haptics scored significantly higher than the control group ($p = 0.004$). In questions 4-8, haptics scored higher again than control ($p = 0.01$). Phase 3: Questions 4-8 (multiple choice), non-haptics scored the highest, followed by the haptic group and then the control.	+/-
Sano	2016	Japan	Crossover trial	7	Medical Patients	Cyberglove II + Kinect	H v NH	Reaching a virtual target with their real intact arm with affected hand virtually controlled (practice attempts)	Reaching a virtual target with their real intact arm with affected hand virtually controlled (non-practice attempts). Done either with haptic feedback or no haptic feedback.	McGill pain sum score before and after the task, questionnaire of perception of affected arm in VR, calculated pain reduction rate, two-sided Wilcoxon signed-rank test	Tactile feedback resulted in more alleviation of deafferentation pain ($p = 0.047$). Average reduction rates were different from 0 under tactile feedback ($p = 0.02$) but not under no tactile feedback ($p = 0.078$). Ownership and agency scores did not significantly differ between the two conditions ($p = 0.13$ and $p = 0.69$, respectively), and their correlation with pain reduction rates was weak and not significant ($p = 0.67$).	-
Girod	2016	USA	Randomized crossover study	10	Medical Residents	Maya + PHANTOM Simulator	H v NH	Familiarization with Maya CAD system and customized haptic system; verbal instruction on both Maya software and customized haptic system	Manipulating virtual bone fragments	Perspectives questionnaire after task; accuracy of virtual fracture repair compared with actual postoperative results	Virtual haptic-simulated repair did not significantly differ from surgical repair ($p = 0.42$). Virtual CAD-simulated repair (non-haptics) differed from surgical repair ($p = 0.02$)	+
Hagelsteen	2017	Sweden	Experimenta l study	20	Medical Students	LapSim® + Simball VR	H v NH	Introduction to LapSimVR on SimFrame and Simball Box; laparoscopic knot tying in Simball VR	LabsimVR training course until proficiency; laparoscopic knot tying	Time to completion, attempts to proficiency, attempts in grasping, attempts in suturing, instrument path length, angular path length, total tissue damage	Haptics completed LapSimVR course faster ($p = 0.002$). Haptics had few attempts in instrument navigation ($p = 0.005$), grasping ($p = 0.017$), and suturing ($p = 0.011$). Haptics had lower instrument total path length ($p = 0.003$) and suturing ($p = 0.030$). Haptics had shorter angular path length ($p = 0.007$). Haptics had lower total maximum damage in instrument navigation ($p = 0.03$) and grasping ($p = 0.019$).	+
Xiong	2017	China	RCT	20	Novices	PHANTOM Device	H v NH	None	Navigate along path of tumor in a VR	Average moving distance, execution time, error length	Haptic group: Average execution time = 1/3 of non-haptic group, Average error distance = 1/2 of non-haptic group (statistical significance not provided).	+/-
Vapenstad	2017	Norway	RCT	30	Medical Students +	LapSim®	H v NH	Passing (based on pilot study by Ahlberg et al.) all component of	Expose and open Calot's triangle, clip and cut the cystic	Expert surgeon rated skills from recorded laparoscopic videos based on depth perception, bimanual dexterity,	Non-haptics group had better laparoscopic video ratings ($p < 0.05$). Non-haptics outperformed haptics in ¾ parameters – perception ($p = 0.025$),	+

					Medical Residents			criterion-based training program based on coordination, clip, applying, lifting, and grasping; theoretical lecture on procedural steps of cholecystectomy; observation of expert surgeon performing procedure in the box model; familiarization and inspection of instruments with researchers	artery and cystic duct and remove the gallbladder from the liver	efficiency, and tissue handing on the Global Operative Assessment of Laparoscopic Skills (GOALS) tool	bimanual dexterity ($p = -0.031$), and efficiency ($p = 0.047$). Tissue handling did not show a statistically significant difference between groups ($p = 0.208$).	
Juo	2017	USA	Crossover study	19	Novices	Da Vinci Robotic System	H v NH	Unspecified	Localization of a hidden tubular structure using the da Vinci surgical robotic system	Mean task completion time for trials under the three different feedback conditions, accuracy in localizing the imbedded tubular structure	Decrease in task completion time did not reach statistical significance with the use of pneumatic normal force feedback alone ($p = 0.762$) but bi-modal haptic feedback use was associated with decreased task completion time ($p = 0.003$). Imbedded tubular structure was best localized by the highest number of subjects with bimodal feedback, followed by pneumatic force feedback alone, and followed by haptic feedback. Increase in discriminatory power was significant only in bi-modal haptic feedback ($p = 0.0009$).	+
Yovanoff	2018	USA	Comparative study	26	Medical Residents	Dynamic Haptic Robotic Trainer (DHRT)	H v NH	Self-efficacy survey; pretest with BluePhantom model; practice needle insertions on assigned training system; practice CVC insertion procedure on a manikin	Needle insertion on manikin	Modified internal jugular catheterization (IJCVC) evaluation form, evaluation form after every trial (participants rate their own performance), self-efficacy survey (pre and post)	Manikin and dynamic haptic robotic trainer (DHRT) training groups improved self-efficacy for CVC insertion skills ($p < 0.001$). DHRT training group increased in confidence in using tactile feedback ($p = 0.034$). The manikin training increased in confidence in placing the needle in the center of the vessel ($p = 0.069$). Manikin group rated their performance higher than the robotic group throughout the trials ($p < 0.001$). The robotic group reported more total errors, angle of insertion errors, final distance errors, and multiple insertion attempt errors ($p < 0.05$). The manikin group reported more errors in pressure or torque during insertion attempts ($p = 0.005$).	+
Dwisaptarini	2018	Thailand	RCT	62	Dentists + Dentistry Students	3D Multilayered Caries Model	H v NH	Caries removal traning with VR simulator vs conventional training with extracted teeth	Minimally invasive caries removal	Performance scores assessed by expert post-training day 5 of minimally invasive caries removal; tooth mass loss, task completion time	The mean post training performance scores were not significantly different between haptics and non-haptics groups ($p = 0.00$). Significant improvements in some areas – superficial dentin ($p < 0.05$), deep dentin/pulpal floor ($p < 0.05$). No differences in tooth mass removed and task completion time after training.	+

Kolarski	2018	USA	RCT	N/A	Medical Residents	Neurotouch	H v NH	Each session on Neurotouch consisted of two practice tasks (sphenoid endoscopy and polypectomy)	Evaluation task was an endoscopic sinus surgery	Performance metrics - quality, efficiency, safety, calculated learning, curve for each task	Endoscopy task: Haptic feedback improved task completion time compared to the non-haptic condition (p < 0.001). There was improvement in task completion time between the first and third attempts, which was sustained during the eighth attempt with haptic feedback (p = 0.05 for the first vs third attempt, p = 0.001 for the first vs eighth attempt). The variance between trainees narrowed with successive practice attempts using haptic feedback. Polypectomy task: No significant difference in task performance between the haptic and non-haptic conditions. Evaluation task: Haptics trended towards better performance, although improvement scores did not reach statistical significance (p = 0.09 for first vs seventh attempt, p = 0.09 for first vs eighth attempt).	+
Francone	2019	USA	Crossover controlled trial	6	Ophthalmologists + Engineers	CHAI3D	H v NH	Introduction and training to perform preretinal membrane peeling tasks x10	Preretinal membrane peeling task	Time, tool tip trajectory, number of tools, retina collisions, distance of penetration, force applied on the retina	Average total tip distance lower without haptics (p = 0.66). Task completion time was significantly shorter with haptics (p = 0.02). Average tool-retina collision force was lower with haptics (p = 0.01). An average of 3.8 retinal whitening episodes (p = 0.18) and 0.33 bleeding episodes (p = 0.25) occurred without haptics per trial.	+
Camara	2019	UK	Crossover controlled trial	20	Physicians	PHANTOM Omni Device	H v NH, Auditory vs Non-Auditory, Visual vs Non-visual	Informed on task; data given to practice ultrasound scanning	Scan the surface of the kidney and as much of the tumour as possible	Participants were given a post survey that included questions about their experience with the feedback category, decision making process, ability to perform intended scans, and their preferred type of feedback; percentageIN (amount of simulated tumor volume within reference boundaries) and totalscanned (percentage of simulated tumor volume failing within boundaries)	Haptics and visual feedback resulted in the best scanning performance. Haptics improved performance in 82.5% cases. Overall scanning performance improved by 81% with any feedback guidance. PercentageIN (case) with haptic feedback was reported as 90.8 and percentageIN (control) with haptics was reported as 82.3.	+
Hagelsteen	2019	Sweden	Randomized Crossover Study	26	Physicians (Surgeons)	LapSim®	H v NH	Oral instructions; grasping and holding with haptics; instructional video on VR and suturing	Suturing attempts	Post-survey evaluating perception and graphical aspect; maximum stretch damage, maximum damage, number of damages	Higher total score in haptic feedback ratings for needle, tissue, and thread (p = 0.008). No difference in graphics scores between settings or groups (p > 0.3 and p = 0.07). No difference in maximum damage and tissue damage.	-
Deng	2020	UK	Crossover controlled trial	10	Novices	VR Dataset	H v NH	None	Measurement of cylinder's length and horizontal and vertical diameter	Participant experience questionnaire	90% found haptics useful in deciding measurement points. 88.9% felt more immersed in VR scene with haptics. A marginal improvement in accuracy with haptics was not significant.	-
Choi	2020	China	Randomized crossover study	15	Novices	Virtual Trackball	Mouse vs H v NH	Oral instructions; video of system operations; familiarization period of operations of virtual trackball with a 2D mouse and 3D haptic device	Manipulate plate model around the bone model changing its position and orientation in 3D	Completion time and placement accuracy; IBM Computer System Usability Questionnaire (CSUQ)	Completion time reduction was significant in haptics (p = 0.008) but not for non-haptics (p = 0.070) while completion time with the mouse remained the same (0.703). Non-haptics remained unchanged, while haptics improved for the harder level (p = 0.014).	+
Chen	2020	USA	Comparative study	10	Medical Residents	Dynamic Haptic Robotic Trainer (DHRT)	H v NH	US-IJCVC placement training compared using DHRT and manikin methods; training based on resident cohort year; proficiency assessment after training	Demographic survey; US-IJCVC assessment; evaluation form and received feedback on their procedural skills	Percent of procedure completed skills on the US-IJCVC, number of insertion attempts, inadvertent arterial puncture, unsuccessful insertion, assistance on procedure, central line experience	No significant impact of training group (DHRT or manikin) on the percent of the total US-IJCVC procedure completed (p = 0.805). Median number of insertion attempts was significantly more than optimal for both DHRT and manikin-trained residents (p = 0.002). No significant effect of training group or central line experience on any of the 23 skills assessed (p = 0.197 to p = 0.998).	+

Benjamin	2021	UK	RCT	8	Medical Students + Physicians + Biomedical Scientists	Fundamental VR	H v NH	Oral instructions; VR calibration test; familiarization with VR and haptics	Drilling the same-sided tibia bone	Pre-survey (demographics); post-survey (perspectives on haptics); plunge distance of drill	No difference in plunge distance of haptic group (t(6) = -1.46; p = 0.1952). No haptics differed in plunge depth (Mean = 79mm, Median = 68mm, SD = 44.23).	+
Huber	2021	Germany	RCT	20	Medical Residents	HaptiVisT (HVT)	H v NH	Oral introduction of study design and tasks; group A trained on cadaver forearm using power drill to insert K-wires; group B performed the same procedure on the HVT simulator with VR training and transparent soft tissue visualization with the option of virtual fluoroscopy for K wire position	K wire fixation on a cadaver forearm without assistance	Total time of the training course, number of drilling attempts of the first and second K-wire, duration of individual drill test	Haptics completed the task faster than non-haptics (p < 0.05). Distance to styloid process larger in non-haptics than haptics (p < 0.05). Protrusion for first K-wire smaller in haptics than non-haptics (p < 0.05). Protrusion of second K- smaller in haptics than non-haptics (p < 0.05). First K-wire position incorrect in 3 cases for non-haptics, all correct for haptics. Second K-wire position incorrect 6 times in non-haptics and 1 time in haptics.	+
Farag	2021	Saudi Arabia	Controlled trial	21	Dentistry Students	Simodont Dental Trainer	H v NH	Orientation session with practical demonstration; second orientation introduced HVRS Simodont dental trainer, and they practiced 20 min/day for 2 weeks; third orientation included a practical demonstration for class I cavity preparation	Cavity preparations	Ratings (total score out of 16 marks), mean time of the cavity preparation after haptic virtual reality simulation (HVRS) training, pulpal floor smoothness, pulpal floor direction, buccal wall direction, lingual wall direction, mesial wall direction, mesial wall smoothness, distal wall smoothness, internal line angle, internal point angle	Decrease in the mean time of cavity preparation after HVRS training (p < 0.001). Increase in the mean of total marks after HVRS training (p = 0.001). Improvement in all evaluation criteria scores after HVRS training.	+
Vamadevan	2022	Denmark	RCT	36	Medical Residents (Surgeons)	LapSim®	H v NH	Proficiency-based laparoscopic simulator training	Time to reach proficiency, instrument path length, instrument angular path length, tissue damage, bleeding, and energy damage	Time to reach proficiency for all tasks, time to reach proficiency on the conventional non-haptic setting after no laparoscopic training, instructor time spent on feedback during training, number of malfunctions, and time spent solving them during the intervention and follow-up test, cumulative time training to proficiency.	Haptics reached proficiency faster during the intervention (p=0.001). Non-haptic group reached proficiency faster during the follow-up test (p<0.001). The haptics group did not improve their time in the follow up (p = 0.22) while the non-haptic group did (p < 0.001). Haptics required less instructor assistance during the intervention (p < 0.001). Time needed for instructor assistance was higher with haptics in the follow-up test (p<0.001). No difference in the cumulated time spent getting instructor-based feedback (p=0.38), number of technical malfunctions (p=0.10), and cumulated time spent reaching proficiency (p=0.42).	-
Awad	2022	Germany	Experimenta l study	25	Orthognathic Surgery Patients	KaVo 3D Orthopanto mographTM	H v NH	Unspecified	Single-jaw orthognathic and bimaxillary surgery	Performance metrics – initial presurgical situation, initial position of the mandible, post operative position of the mandible defined by virtual occlusal adjustment, postoperative position of the mandible defined by conventional adjustment, maxilla shielded, and a measurement in X-, y-, and z- axes.	No significant differences between haptics vs non-haptics in the postsurgical position of the mandible (p = 0.580 to 0.713).	+/-
Gani	2022	UK	RCT	31	Medical Residents	Fundamental Surgery VR	H v NH	Haptics and non-haptics group completed three drilling holes through a simulated tibia bone model using a surgical drill in an immersive VR training module with the goal to practice drilling	Drilling 3 holes through a Tibial Sawbone model in an ex vivo environment	Performance metrics – plunge gap distance, drilling time, objective structured assessment of technical skills (OSATS) ratings, and plunge depth of the drill beyond the target tissue<br>Performance metrics – plunge gap distance and drilling time.	The haptics group achieved a smaller plunge gap distance (p = 0.012), longer mean drilling time (p = 0.027), higher combined OSATS scores (p = 0.0006), higher ratings in the procedural flow domain (p = 0.029), higher scores in the instrument handling domain (p = 0.053), and more participants in the haptics group achieved a learning plateau compared to the non-haptics group (p = 0.01).	+

								through both cortices of the bone while minimizing drill plunge depth beyond the far side of the bone to prevent tissue damage.<br>The haptic group received haptic feedback upon use and manipulation of the drill, while the non-haptic group relied on visual and auditory feedback alone.		Objective structured assessment of technical skills (OSATS) where expert consultant surgeons rated video performances based on time, motion, instrument handling, procedural flow, overall performance, safe drill (achieving plunge gap of <5 mm in at least 2/3 attempts), learning curve plateau, and participant feedback questionnaire.	The haptics group showed a significant difference with 40% of participants achieving a safe drill depth in 2/3 attempts, while none in the non-haptics group achieved this (p = 0.02).<br>Both groups had similar ratings in the time/motion and overall performance domains (p = 0.053).<br>In the participant questionnaire, more participants in the haptics group reported that instruments felt and sounded more realistic than the non-haptics group (p = 0.006 and p = 0.03, respectively), both groups reported high ratings for simulation enjoyment and perceived education value, both groups agreed that the simulation improved theoretical knowledge and instrument handling, the haptics group rated the role of simulation in recognizing when to stop drilling significantly higher than the non-haptic group (p = 0.039, and both groups expressed positive views on the use of simulation in surgical training and its potential to improve clinical outcomes.	
Scott	2022	Canada	Crossover study	7	Physicians (Surgeons)	Physics-driven MI Spinal Fusion Surgical Simulator	H v NH	None	Minimally invasive spinal fusion procedure on a cadaver torso involving accessing and removing the intervertebral disc followed by inserting a bone graft and cage	Wilcoxon's non-parametric rank-sum test was employed using MATLAB to examine the connection between task completion time and the overall procedure duration in both cadaver and simulator trials.<br>Participant feedback through a questionnaire.	The Wilcoxon test detected statistically significant decreased time taken to gain access to the surgical area (p = 0.033) and the time taken to attach the surgical port, perform a facetectomy, and use the powered burr, as well as the total procedural time (p = 0.017) in the haptics group.<br>Statistical significance was observed of time spent using the surgical probe and the powered burr (p = 0/017).<br>The median score allocated to 94% of the questions was above the defined limit of sufficient validity (≥3/5) in the questionnaire.	+
Domes	2023	USA	RCT	18	Medical Students	AAAOS/OTA Simulator	H v NH	The trained group completed 9 simulator-based modules with the final assessed task of placing 3 wires in a desired configuration; untrained group received a hands-on simulator orientation	Pin placement in whole-body cadaver hips using fluoroscopic imaging	Distance to ideals for the middle inferior, superior anterior, and superior posterior aspects of the femoral neck, and to the articular surface of the femoral head and the distance from the lower border of the lesser trochanter to the most inferior pin.<br>Primary evaluator evaluation of post-procedure CT scans and final AP and lateral fluoroscopic images to determine whether triangle construct was achieved.	The haptics trained group was significantly better at achieving an inverted triangle construct compared to the untrained group (p = 0.05).<br>No statistically significant differences were observed between the haptics trained and untrained groups regarding the number of AP or lateral view images obtained or fluoroscopic time.<br>No statistically significant differences were found between the haptics trained group and untrained groups related to the distance from the tip of the wire to the femoral head articular surface as shown by the CT in the axial and coronal planes.<br>There were statistically significant differences favouring the haptics trained group for the angle between the inferior and superior posterior pin (p < 0.01), angle between the inferior and superior anterior pin (p = 0.02), and superior anterior pin distance to mid-neck (p < 0.01).	+
Philip	2023	Switzerland	RCT	14	Dentistry Students	HVRS (Haptic Virtual Reality Simulator)	H v NH	Participants practiced and performed the pulpotomy procedures using haptics in one group, and convention simulation environment in the non-haptics group.	Primary molar pulpotomy procedure	Performance metrics – procedural time, number of instructor prompts, and student perceptions which included questions on the realism of images displayed on the monitor, tactile sensation, and texture/hardness of dental tissue, the impact of psychomotor skills and confidence, and whether training can replace or supplement conventional pre-clinical training.	The haptics group had non-significant higher access outline scores (p = 0.67), non-significant higher deroofing skills (p = 0.37), non-significant different in access outline prompts (p = 1.0), non-significant deroofing prompts, non-significantly lower procedural time, and no difference in the number of instructor prompts.<br>Student perceptions – 72% of students agreed/strongly agreed that images of teeth, pulp chamber, and instruments displayed on the monitor looked realistic, mixed responses on the realism and tactile sensations and texture/ hardness of	+/-

	dental tissues, 443% agreed/strongly agreed that the tactile force feedback felt realistic, 50% indicated that they could not differentiate between enamel and dentine texture/hardness on the simulator, 64% of students agreed/strongly agreed that training on the haptics device improved their fine motor dental skills and confidence in performing the pulpotomy procedure, a significant majority (86%) disagreed/strongly disagreed with the notion that haptics training could replace conventional pre-clinical training on typodont teeth for the pulpotomy procedure, 57.1% of students expressed interest in having more haptic session for preclinical pediatric pulp procedures, and student responses from open-ended questions indicated that the main benefits of haptic training was increased practice opportunities, better visualization, and improved fine motor skills.

Note. Findings: ++ very strong, + strong, +/- neutral or ambiguous, - weak, - very weak

Table 2.

Assessed Parameters, Including Objective (time, path length, movements, etc.) and Subjective (ease of use, preference, etc.) Tasks

Tasks being Assessed	N
**Surgical Tasks**	101
Time Measurement	20
Subjective Assessment	17
Objective Assessment	32
Instrument Dexterity	27
Orthopedic Specific Tasks	2
Surgical Scanning	3
**Dental Specific Tasks**	31
Cavity inspection	10
Implantology-related drilling outcomes	12
Molar preparation	3
Molar insertion task	6
**Non-Surgical Tasks**	12
Time Measurement	1
Subjective Assessment	9
Objective Assessment	2

Table 3.

Conditions Favored

Article	Assessed Tasks	Condition favored	More context (if necessary)
Francone, 2019	Time-to-task completion	Favoured haptics	
	Total length of the tool tip trajectory	Favoured haptics	
	Number of tool-retinal collisions	Favoured haptics	
	Distance of penetration into the retina	Favoured haptics	
	Force applied on the retina along the vertical axis	Favoured haptics	
Deng, 2020	A questionnaire comparing their experience of haptic-enabled test condition and haptic-disabled test condition	Favoured haptics	
Vamadevan, 2022	Time spent to reach the predefined proficiency level (intervention)	Favoured haptics	
	Time spent to reach the predefined proficiency level (follow-up)	Did not favour haptics	
	Instructor assistance during intervention	Favoured haptics	During the intervention, the haptic group required significantly less instructor assistance than the non-haptic group (p< 0.001)
	Instructor assistance during follow-up	Did not favour haptics	The time needed for instructor assistance was significantly higher for the haptic group compared to the non-haptic group (p<0.001).

	Instructor assistance between intervention and follow-up	Mixed	The haptic group required significantly more feedback from the instructor for the follow-up test than the assistance needed during the intervention phase (*p* < 0.001; e.g., once haptic group transitioned to non-haptic they required more instructor assistance). In contrast, the non-haptic group used significantly less help from the instructor during the follow-up phase (*p* < 0.001).
	Number of malfunctions and time spent solving them during the intervention and the follow-up test	Neither condition	
	Cumulative time training to proficiency	Neither condition	
Camara, 2019	Post-survey on condition preference	Favoured haptics	
	PercentageIN (Amount of simulated tumor volume within the boundaries of the reference volume)	Favoured haptics	
	TotalScanned (Percentage of simulated tumor volume falling within the reference tumor volume)	Favoured haptics	
	BinaryCount (Difference in scanning between haptic modalities)	Favoured haptics	
Hagelsteen, 2019	Post-survey	Favoured haptics	
	Ratings of 5 graphical aspects	Neither condition	
	Maximum stretch damage (%)	Favoured haptics	
	Maximum damage(mm)	Neither condition	
	Number of damages to the tissue	Neither condition	
Choi, 2020	Completion time	Favoured haptics	
	Placement accuracy	Favoured haptics	However, Accuracy in mode M significantly improved over four sessions, particularly for hard levels (p = 0.012 for easy level, p = 0.008 for hard level), while mode HNF

			showed no significant change and mode HF improved for hard levels (p = 0.014).
	IBM Computer System Usability Questionnaire	Favoured haptics	
Benjamin, 2021	Plunge distance of the drill	Neither condition	
Hagelsteen, 2017	Time to completion	Favoured haptics	
	Attempts to reach proficiency	Favoured haptics	
	Attempts in grasping task	Favoured haptics	
	Attempts in suturing task	Favoured haptics	
	Instrument path length	Neither condition	
	Angular path length	Favoured haptics	
	Total tissue damage	Favoured haptics	
	Performance pre- and post the LapSim® course	Neither condition	
Erolin, 2016	Anatomy test for phase 1	Favoured haptics	
	Anatomy test for phase 2	Favoured haptics	
	Anatomy test for phase 3	Neither condition	In questions 1 the non-haptic condition scored highest. In question 2 and 3 the haptic condition scored highest. In questions 4-8, the non-haptic group scored the highest, followed by the haptic group and then the control. However, only the control vs. non-haptic group had a significant difference (p = 0.007)
Gosling, 2005	Post-fracture reduction angular deformity	Neither condition	
	Fracture reduction time	Neither condition	
	Image control (number of images used)	Favoured Haptic	
	Post-fracture reduction fracture gap distraction (lower is better)	Favoured Haptic	

Kim, 2004	Training effectiveness	Favoured haptics	
Lyu, 2013	Normal path error	Favoured haptics	
	Operating time	Favoured haptics	
Panait, 2009	Task completion time	Favoured haptics	
	Instrument path lengths	Favoured haptics	
	Right- and left-hand errors	Favoured haptics	This result is only for difficulty 1. No significant difference was achieved at level 2 and 3.
	Grasping tension	Favoured haptics	
Salkini, 2010	Simulator's reported accuracy (%)	Neither condition	
	Economy of movement of each hand (%)	Neither condition	Videogame players tended to have more economic and faster hand movement than their colleagues in both groups (significant for the dominant hand only).
	Average speed of each hand (cm/s)	Neither condition	
Sano, 2016	A questionnaire for sense of reality	Favoured haptics	
	A questionnaire for pain intensity	Favoured haptics	
	Elbow angles	Favoured haptics	
	Average of reduction rates	Favoured haptics	
Sengul, 2013	Mean reaction times	Favoured haptics	
	Error rates per condition	Neither condition	
	Crossmodal congruency effect: the performance difference (reaction time or accuracy) between incongruent and congruent visuo-tactile stimulations.	Favoured haptics	
Thompson, 2011	Total time	Did not favour haptics	
	Efficiency of cautery	Neither condition	
	Number of right instrument movements	Did not favour haptics	

	Speed of right instrument	Favoured haptics	
	Number of left instrument movements	Favoured haptics	
	Speed of left instrument	Neither condition	
	Number of trials needed to reach the criteria for the basic skills tasks	Neither condition	
	Performance of haptic and non-haptic cholecystectomy trials	Neither condition	
	Learning curve	Neither condition	
Vapenstad, 2013	A questionnaire designed to collect information and opinions from participants regarding two key aspects: their background and their perception of two handles.	Did not favour haptics	Most surgeons believe that grips with haptic feedback on VR simulators are important and that grips that provide a sense of tissue hardness must be realistic. However, most surgeons also believe that grips without haptic feedback feel the most realistic and perform the best in use.
Xiong, 2017	Average moving distance	Favoured haptics	
	Average execution time	Favoured haptics	
	Average error length	Favoured haptics	
Strom, 2006	BasIQ general cognitive ability test	Neither condition	
	Mental Rotation Test A	Neither condition	
	The Flow questionnaire	Neither condition	
	The Mental Effort questionnaire on the Borg CR10 scale	Neither condition	
	Performance variables (time, movement economy, tool–tool collision error, shaft–target collision error, and instrument, diathermy, and pedal error) after 1h of training	Neither condition	
	Performance variables after 2h of training	Favoured haptics-first group	
	Changes of performance over time	Favoured haptics-first group	
Cao, 2007	Time-to-task completion	Favoured haptics	

	Number of errors	Favoured haptics	
	Total number of math problems completed	Favoured haptics	
Huber, 2021	The total time of the training course	Non-haptic	
	The number of drilling attempts of the first and second K-wire	Neither condition	
	The duration of individual drill test	Favoured haptics	
	The distance between the optimal and actual entry point at the styloid radial process	Favoured haptics	
	The fluoroscopy image evaluation	Favoured haptics	
Chen, 2020	Percentage of ultrasound-guided Internal Jugular Central Venous Catheterization procedure completed	Neither condition	
	Insertion attempts	Neither condition	Both DHRT- (haptics) and manikin-trained (non-haptics) required a median number of insertion attempts more than the optimal one needle attempt
	Inadvertent arterial puncture	Neither condition	
	Unsuccessful insertion	Neither condition	
	Assistance on procedure	Neither condition	
	Assistance required on procedure predicted by central line experience (predictor variable)	Neither condition	
Farag, 2021	Mean time of cavity preparation	Favoured haptics	
	Pulpal floor smoothness	Favoured haptics	
	Pulpal floor direction	Favoured haptics	
	Buccal wall direction	Favoured haptics	
	Lingual wall direction	Favoured haptics	
	Mesial wall direction	Favoured haptics	
	Mesial wall smoothness	Favoured haptics	
	Distal wall smoothness	Favoured haptics	
	Internal line angle	Favoured haptics	
	Internal point angle	Favoured haptics	

	Psychomotor skills evaluation tool	Favoured haptics	
Yovanoff, 2018	Pre- and post-central line self-efficacy survey	Mixed results	Manikin (non-haptics) group, all survey questions were significantly improved from pre- to post-test. DHRT (haptics) group improved from pre- to post-test except for "location of needle on ultrasound image"
	CVC insertion self-efficacy	Mixed results	Both manikin (non-haptics) and DHRT (haptics) groups showed improvements in CVC insertion self-efficacy over the course of training
	Post-trial self-ratings of performance	Did not favour haptics	
	Errors during training relating to force	Favoured haptics	The manikin group reported more errors in pressure or torque during insertion attempts ($p = 0.005$).
	General errors during training	Did not favour haptics	The robotic group reported more total errors, angle of insertion errors, final distance errors, and multiple insertion attempt errors ($p < 0.05$)
	Accurate prediction of objective performance score	Favoured haptics	
Balci, 2014	Duration of operations	Neither condition	
	OSATS assessment criteria – tissue	Neither condition	
	OSATS assessment criteria – duration of operations and manipulations	Neither condition	
	OSATS assessment criteria – instrumental experience	Neither condition	
	OSATS assessment criteria – safety of manipulations	Neither condition	
	OSATS assessment criteria – use of an assistant	Neither condition	
	OSATS assessment criteria – flow of operations	Neither condition	
	OSATS assessment criteria – accuracy of operative technique	Neither condition	

Chmarra, 2008	Time, path length, and depth perception (ball task)	Neither condition	
	Time, Path Length, and Depth Perception (Ring Task)	Neither condition	
	Time, Path Length, and Depth Perception (Elastic Band Task)	Mixed	Box (haptic)-VR (non-haptic) group performed 50% faster than VR-box ($p < 0.01$) in the elastic band task. Box-VR group had shorter path length for left (60%, $p < 0.01$) and right (55%, $p < 0.01$) instrument. Box-VR group had shorter depth perception for left (65%, $p < 0.01$) and right (50%, $p < 0.01$) instruments. VR trainer showed significant difference only for right instrument: path length 70% shorter ($p < 0.01$), depth perception 65% shorter ($p < 0.02$).
Cohen, 2006	Objective competence (colonoscopy)	Favoured haptics	
	Subjective competence (colonoscopy)	Favoured haptics	
	Patient discomfort (colonoscopy; measured by proctor)	Neither condition	
	Blocks to reach 90% competency (colonoscopy)	Neither condition	
Dwisaptarini, 2018	Face Validity of Minimally Invasive Caries Removal Simulator	Favoured haptics	
	Minimally Invasive Caries Removal Performance	Favoured haptics	
	Tooth mass loss	Neither condition	
	Task Completion Time	Neither condition	
Gerovich, 2004	Reduced error in skin, fat, and muscle	Favoured haptics	
	Layer transition detection	Favoured haptics	
	Reduction of use of static display of tissue layers	Favoured haptics	

Girod, 2016	Mandibular fracture reduction	Favoured haptics
	Measurement of accuracy	Favoured haptics
	User experience assessment	Favoured haptics
Hedman, 2006	Instrument navigation	Favoured haptics
	Manipulate and diathermy	Neither condition
Hochman, 2015	Experience survey – Mechanical properties (cortical, trabecular, and osseous realism) of haptics vs non-haptics	Did not favour haptics
	Experience survey – Anatomical features	Neither condition
	Learning cortical mastoidectomy	Did not favour haptics
	Learning posterior tympanotomy/facial recess approach to the middle ear	Did not favour haptics
	Ease of use	Neither condition
Hogle, 2008	Depth perception in cholecystectomies in pigs	Favoured haptics
	Bimanual dexterity, efficacy, tissue handling, and autonomy in cholecystectomies in pigs	Neither condition
Joseph, 2014	Mean position deviation improved (1st vs 4th test)	Favoured haptics
	Accuracy of 8th trial vs 1st trial	Favoured haptics
	Drilling depth improvement	Favoured haptics
	Virtual guidance reduction of cortical perforations	Favoured haptics
	Decreased parameters after trials	Favoured haptics
	Mean buccolingual angle	Neither condition
	Mean mesiodistal angle deviation	Neither condition
	Mean drilling depth	Neither condition
	Centering error for point of impact	Favoured haptics
	Quality of implant site preparation	Favoured haptics

Koo, 2015	Game-like features increasing fun and interest	Favoured haptics	
	Ease of use	Favoured haptics	
	Device usefulness at home	Favoured haptics	
	Ease of real-life task performance after sing simulator and subjective evaluation of manual dexterity	Mixed	67% disagreed or strongly disagreed that it was easier to perform the task after the simulation exercises or that haptics improved manual dexterity for handling the handpiece. However, 59% disagreed or strongly disagreed that the amount of time engaged with haptics was sufficient to evaluate effectiveness
Kulscar, 2013	Written test on technique of spinal anesthesia score	Neither condition	
	Global rating scales during simulator training	Neither condition	
	Clinical testing assessment	Favoured haptics	
	Video assessment of clinical performance	Neither condition	
	Task specific checklist agreement (video assessments)	Neither condition	
LeBlanc, 2013	Itemized checklist on fixation of ulna task	Favoured haptics	
	Global rating scale score	Favoured haptics	
	Time to task completion	Did not favour haptics	
	Subjective evaluation of simulators	Did not favour haptics	Residents found that the virtual simulator needed further improvement ($p < 0.05$) and that they would prefer the sawbones (non-haptic) simulator if given the choice ($p < 0.05$).
Liu, 2013	Temporal bone anatomy comprehension improvement	Favoured haptics	
	Written examination on temporal bone anatomy and dissection improvement	Favoured haptics	

Park, 2015	Arc resection error	Favoured haptics	
	Resection start error	Favoured haptics	
	Resection End error	Favoured haptics	
	Over-resection volume error	Favoured haptics	Haptics resulted in less over-resection (mean volume error ($p < 0.01$).
	Maximum resection depth	Neither condition	
	Cutting time	Favoured haptics	
Vapenstad, 2017	Laparoscopic video rating – depth perception, manual dexterity, and efficiency	Did not favour haptics	
	Tissue handling	Neither condition	
Zhou, 2012	Learning curve analysis	Favoured haptics	Non-haptics reached performance plateau by the 6th session, while the haptics group reached it earlier, by the 5th session
	Learning rate comparison	Neither condition	
	Time to task completion across all trials	Favoured haptics	
	Best trial speed	Favoured haptics	
	Variance in task completion time per session	Favoured haptics	
	Equivalence by knots of subjects	Neither condition	
Chao, 2015	Mean image quality score	Favoured haptics	
	Rate of satisfactory images taken	Neither condition	
	Feedback survey – confidence, speed, images, and orientation	Neither condition	All trainees found the simulator to improve confidence in scanning real patients, their capacity to produce adequate images, and their capacity to get oriented. All also said that conventional teaching remains useful in addition to virtual simulation teaching. Slightly more trainees in the control group (non-haptics) believed the simulator would

			improve scanning speed compared to the simulator (haptics) group.
	Usefulness of conventional training	Neither condition	
Bouhelal, 2014	Basic task 5 mean total simulator time	Favoured haptics	
	Basic task 6 mean total simulator time	Favoured haptics	
	Procedural task 3 mean total simulator time	Favoured haptics	
	Procedural task 4 mean total simulator time	Favoured haptics	
	Full learning curve proficiency in mean total simulator time	Favoured haptics	
Juo, 2017	Decrease in task completion time (pneumatic normal haptic feedback alone)	Neither condition	
	Decrease in task completion time (bi-modal haptic feedback which warns users of excessive force)	Favoured haptics	
	Discriminatory power (localization of critical vascular or neural structures imbedded within soft tissue)	Favoured haptics	Increase in discriminatory power was significant only in bi-modal haptic feedback ($p = 0.0009$).
Kolarski, 2018	Endoscopy task – average time to completion, overall score, and narrowing of variance between trainees	Favoured haptics	
	Polypectomy task (average scores)	Neither condition	
	Evaluation task scores	Neither condition	
Award, 2022	The initial (presurgical) positions of the mandible	Neither condition	
	The final (postsurgical) positions of the mandible	Neither condition	
Domes, 2023	Fluoroscopic time	Neither condition	
	number of AP	Neither condition	
	lateral view images	Neither condition	
	pin distance to ideals for the middle inferior, superior anterior, and superior posterior aspects of the femoral neck	Favoured haptics	
	pin distance to the articular surface of the femoral head	Neither condition	

	distance from the lower border of the lesser trochanter to the most inferior pin	Favoured haptics	
	Evaluation of the inverted triangle construct (post procedure CT scans, final AP and lateral fluoroscopic images)	Favoured haptics	
Gani, 2022	Benchtop drilling time	Did not favour haptics	
	A pre-procedure questionnaire assessing various aspects, including hand dominance, level of operative experience	Neither condition	
	A post-procedure questionnaire aiming to gather their feedback on the study and its utilization of haptic feedback simulation	Favoured haptics	
	Plunge depth distance	Favoured haptics	
	Learning curve	Favoured haptics	
	Safe drill attempts ((<5 mm plunge gap in at least 2 out 3 attempts))	Favoured haptics	
	Objective Structured Assessment of Technical Skill rating on video performance of benchtop drilling	Favoured haptics	
Philip, 2023	Performance score of access outline and pulp chamber deroofing steps	Neither condition	
	The time taken to complete the two pulpotomy procedural steps on the plastic primary tooth	Neither condition	
	Number of times he/she may have requested instructor help	Neither condition	
	A survey questionnaire recording participants' experiences and perceptions	Mixed results	An overwhelming majority of students (86%) disagreed or strongly disagreed with the statement that HVRS training can replace conventional pre-clinical training on typodont teeth for the pulpotomy procedure, with most suggesting that HVRS may be used as an adjunct to the conventional simulation training.<br>Most of the students also preferred to experience the HVRS training after the conventional simulation training.

Stott, 2022	A questionnaire measuring the face and content validity of analog surgical instruments.	Haptic

Table 4.

MERSQI Results

Domain	MERSQI Item	Studies No. (%)	Score		Mean (SD)	
			Item	Maximum Domain	Item	Domain
Study design	1. Study design			3	2.18 (0.79)	2.18 (0.79)
	Single group cross-sectional or single group post-test only	8 (15.7%)	1			
	Single group pretest and post-test	10 (19.6%)	1.5			
	Nonrandomized, 2 groups	11 (21.6%)	2			
	Randomized controlled trial	22 (43.1%)	3			
Sampling	2. No/ of institutions studied			3	0.55 (0.21)	1.40 (0.40)
	1	48 (94.1%)	0.5			
	2	1 (2.0%)	1			
	>2	2 (3.9%)	1.5			
	3. Response rate, %				0.85 (0.48)	
	Not applicable	0 (0.0%)				
	<50 or not reported	33 (64.7%)	0.5			
	50-74	0 (0%)	1			
	≥ 75	18 (35.3%)	1.5			
Type of data	4. Type of data			3	2.88 (0.48)	2.88 (0.48)
	Assessment by study participant	3 (5.9%)	1			
	Objective measurement	48 (94.1%)	3			
Validity of evaluation instrument	5. Internal structure			3	0.75 (0.44)	1.76 (0.49)
	Not applicable	11 (21.6%)				
	Not reported	10 (19.6%)	0			
	Reported	30 (58.8%)	1			
	6. Content				0.71 (0.46)	
	Not applicable	10 (19.6%)				
	Not reported	12 (23.5%)	0			
	Reported	29 (56.9%)	1			
	7. Relationships to other variables				0.30 (0.46)	
	Not applicable	11 (21.6%)				
	Not reported	28 (54.9%)	0			
	Reported	12 (23.5%)	1			
Data analysis	8. Appropriateness of analysis			3	1.00 (0.00)	2.90 (0.50)
	Data analysis inappropriate For study design or type of data	0 (0.0%)	0			
	Data analysis appropriate for study design or type of data	51 (100.0%)	1			
	9. Complexity of analysis				1.90 (0.30)	
	Descriptive analysis only	5 (9.8%)	1			
	Beyond descriptive analysis only	46 (90.2%)	2			
Outcomes	10. Outcomes			3	1.58 (0.40)	1.58 (0.40)
	Satisfaction, attitudes, perceptions, opinions, general facts	4 (7.8%)	1			
	Knowledge, skills	41 (80.4%)	1.5			
	Behaviours	3 (5.9%)	2			
	Patient/health care outcomes	3 (5.9%)	3			
**Total score**				**18**		12.82 (1.59)

Table 5.

CASP RCT Standard Checklist Results

	Section A: Is the basic study design valid for a randomised controlled trial?			Section B: Was the study methodologically sound?					Section C: What are the results?			Section D: Will the results help locally?	
**Study**	**Did the study address a clearly focused research question?**	**Was the assignment of participants to the interventions randomized?**	**Were all participants who entered the study accounted for at its conclusion?**	**Were the participants "blind" to the intervention they were given?**	**Were the investigators "blind" to the intervention they were giving to the participants?**	**Were the people assessing/analysing outcome/s 'blinded'?**	**Were the study groups similar at the start of the randomized controlled trial?**	**Apart from the experimental intervention, did each study group receive the same level of care (that is, were they treated equally)?**	**Were the effects of intervention reported comprehensively?**	**Was the precision of the estimate of the intervention or treatment effect reported?**	**Do the benefits of the experimental intervention outweigh the harms and costs?**	**Can the results be applied to your local population/in your context?**	**Would the experimental intervention provide greater value to the people in your care than any of the existing interventions?**
LeBlanc, 2013	Yes	Yes	Yes	No	No	Yes	Yes	Yes	Yes	Yes	Yes	Yes	Yes
Balci, 2014	Yes	Yes	Yes	Yes	Yes	No	Yes	Yes	Yes	Yes	Yes	Yes	Yes
Benjamin, 2021	Yes	Yes	Yes	No	No	Yes	Yes	Yes	Yes	No	Yes	Yes	Yes
Chao, 2015	Yes	Yes	Yes	No	No	Yes	Yes	Yes	Yes	Yes	Yes	Yes	Yes
Cohen, 2006	Yes	Yes	No	No	No	Yes	Yes	Yes	Yes	Yes	Yes	Yes	Yes
Domes, 2023	Yes	Yes	Yes	No	No	Yes	Yes	Yes	Yes	Yes	Yes	Yes	Yes
Dwisaptarini, 2018	Yes	Yes	Yes	No	No	Yes	Yes	Yes	Yes	Yes	Yes	Yes	Yes
Gani, 2022	Yes	Yes	Yes	Yes	Yes	Yes	Yes	Yes	Yes	Yes	Yes	Yes	Yes
Hogle, 2008	Yes	Yes	Can't tell	No	No	No	Yes	Yes	Yes	No	Yes	Yes	Yes
Huber, 2021	Yes	Yes	Yes	No	No	Yes	Yes	Yes	Yes	Yes	Yes	Yes	Yes
Joseph, 2014	No	Yes	Can't tell	No	No	Yes	No	Yes	Yes	Yes	Yes	No	Yes
Kolarski, 2018	Yes	Yes	Yes	No	No	Can't tell	Can't tell	Yes	Yes	Yes	Yes	Yes	Yes
Koo, 2015	Yes	Yes	Can't tell	No	No	Can't tell	Yes	Yes	Yes	Yes	Yes	Yes	Yes
Kulscar, 2013	Yes	Yes	Yes	No	Yes	Yes	Yes	Yes	Yes	Yes	Yes	Yes	Yes
Liu, 2013	No	Yes	Yes	No	Can't tell	Can't tell	Yes	Can't tell	Yes	No	Yes	No	Yes
Philip, 2023	Yes	Yes	Yes	No	No	Yes	Yes	Yes	Yes	Yes	Yes	Yes	Yes
Salkini, 2010	Yes	Yes	Yes	No	No	No	Yes	Yes	Yes	Yes	Yes	Yes	Yes
Thompson, 2011	No	Yes	Yes	No	No	No	Yes	Yes	Yes	Yes	Yes	No	Yes
Vamadevan, 2022	No	Yes	No	No	No	No	Yes	Yes	Yes	Yes	Yes	No	Yes
Xiong, 2017	Yes	Yes	Yes	No	No	No	Can't tell	Yes	Yes	No	Yes	Yes	Yes
Yovanoff, 2018	Yes	Yes	Yes	No	No	No	No	Yes	Yes	Yes	Yes	Yes	Yes
Zhou, 2012	Yes	Yes	Yes	No	No	No	Can't tell	Yes	Yes	Yes	Yes	Yes	Yes

Table 6.

JBI Critical Appraisal Checklist for Quasi-experimental Studies Results

Study	Is it clear in the study what is the 'cause' and what is the 'effect' (i.e. there is no confusion about which variable comes first)?	Were the participants included in any comparisons similar?	Were the participants included in any comparisons receiving similar treatment/care, other than the exposure or intervention of interest?	Was there a control group?	Were there multiple measurements of the outcome both pre and post the intervention/exposure ?	Was follow up complete and if not, were differences between groups in terms of their follow up adequately described and analyzed?	Were the outcomes of participants included in any comparisons measured in the same way?	Were outcomes measured in a reliable way?	Was appropriate statistical analysis used?
Erolin, 2016	Unclear	Yes	Yes	Yes	Unclear	Not applicable	Yes	Unclear	Yes
Chen, 2020	Yes	No	Yes	Yes	Yes	Not applicable	Yes	Unclear	Yes
Awad, 2022	Yes	Unclear	Yes	Yes	Not applicable	Not applicable	Not applicable	Yes	Yes
Bouhelal, 2014	Unclear	Unclear	Unclear	Yes	Not applicable	Not applicable	Unclear	Yes	Yes
Farag, 2021	Yes	Yes	Yes	Yes	Yes	Yes	Yes	Yes	Yes
Kim, 2004	Yes	Yes	Yes	Yes	Yes	Yes	Yes	Yes	Yes
Hagelsteen, 2017	Yes	Yes	Yes	Yes	Yes	Yes	Yes	Yes	Yes
